# A protective hybrid protein vaccine informed by mapping major histocompatibility class II epitopes in *Cryptococcus neoformans* chitin deacetylases that stimulate CD4^+^ T-cell responses

**DOI:** 10.1128/mbio.02176-26

**Published:** 2026-10-14

**Authors:** Charles A. Specht, Lorena V. N. Oliveira, Diana Carlson, Maureen M. Hester, Satya Sahu, Gabriel Kristian Pedersen, Stuart M. Levitz

**Affiliations:** 1 Department of Medicine, University of Massachusetts Chan Medical School 164186, Worcester, Massachusetts, USA; 2 Center for Vaccine Research, Statens Serum Institut 4326, Copenhagen, Denmark; Instituto Carlos Chagas, Curitiba, Brazil

**Keywords:** fungal vaccines, epitope mapping, peptide library, immunoinformatics, *Cryptococcus neoformans*

## Abstract

Infections due to *Cryptococcus* species are estimated to cause over 100,000 deaths a year. No cryptococcal vaccines are approved for human use. We have shown that vaccine formulations consisting of *C. neoformans* chitin deacetylase 2 (Cda2) and Cda1 adjuvanted with Cationic Adjuvant Formulation 01 (CAF01) protect mice against experimental cryptococcosis by a mechanism dependent upon CD4^+^ T cells. Here, using overlapping peptide libraries, we mapped epitopes in Cda2 and Cda1 that stimulated interferon-gamma (IFNγ) release from splenocytes of C57BL/6 and DR4 mice immunized with these proteins. DR4 mice lack mouse major histocompatibility class II (MHC-II) proteins and express chimeric MHC-II proteins with the specificity of the human HLA-DR allele, HLA-DRB1*04:01. Experimental results were then compared with MHC-II binding predictions using the Immune Epitope Database NetMHCIIpan 4.1 BA MHC-II epitope prediction tool. Unique epitopes in Cda2 and Cda1 were discovered for each mouse strain, some of which were not predicted to bind well to MHC-II. CD4^+^ T cells were responsible for cytokine release as IFNγ production was lost if CD4^+^ T cells were depleted from the splenocyte population. Finally, we tested a hybrid recombinant protein that consisted of portions of Cda2 and Cda1 containing the MHC-II/CD4^+^ T-cell epitopes that induce CD4^+^ T cells in DR4 mice. When administered as a CAF01-adjuvanted vaccine, the hybrid protein protected DR4 mice from an otherwise lethal *C. neoformans* pulmonary challenge. These results provide a proof of principle regarding the utility of MHC-II/CD4^+^ T-cell epitope mapping in cryptococcal vaccine development.

**IMPORTANCE:**

The development of vaccines to prevent cryptococcosis, a fungal disease which causes over 100,000 deaths annually, is a public health priority. We have demonstrated that adjuvanted vaccines containing two *Cryptococcus neoformans* protein antigens, chitin deacetylase (Cda)2 and Cda1, protect mice against experimental cryptococcosis by a mechanism dependent upon CD4^+^ T cells. In this study, we determined which 15-amino-acid peptides of Cda2 and Cda1 stimulate CD4^+^ T cells in C57BL/6 and DR4 mice; the latter mouse strain has human major histocompatibility class II (MHC-II) specificity. We then synthesized a small hybrid protein containing those portions of Cda2 and Cda1 that strongly stimulated CD4^+^ T-cell responses in DR4 mice. As an adjuvanted vaccine, the hybrid Cda2/1 protein protected DR4 mice from a *C. neoformans* pulmonary challenge. Thus, CD4^+^ T-cell/MHC-II epitope mapping experimentally validated the design of a protective multi-epitope protein for use in cryptococcal vaccines.

## INTRODUCTION

Infections due to encapsulated fungi belonging to the *Cryptococcus neoformans* and *C. gattii* species complexes are estimated to kill over 100,000 people annually (1). The vast majority of cryptococcosis cases occur in people with impaired CD4^+^ T-cell function due to factors such as advanced HIV infection, receipt of immunosuppressing medications, and hematological malignancy (2). Most infections are thought to originate following inhalation of airborne fungi; in the absence of effective host defenses, central nervous system dissemination can ensue.

There are no approved cryptococcal vaccines for use in humans. Vaccine formulations consisting of attenuated *C. neoformans* strains and adjuvanted subunit formulations protect mice against experimental cryptococcosis (reviewed in references 3–5). Critical for the development of protective subunit vaccines is antigen and adjuvant discovery. Regarding antigens, we have extensively studied two promising protein candidates, chitin deacetylase 2 (Cda2) and Cda1. When adjuvanted in glucan particles or with cationic adjuvant formulation 01 (CAF01), Cda2 and Cda1 protect BALB/c, C57BL/6, and DR4 mice from an otherwise lethal *C. neoformans* pulmonary challenge (6–11). The importance of T helper (Th) cells is suggested by data showing protection is lost in mice that lack CD4^+^ T cells or interferon-gamma (IFNγ). Moreover, mice that are vaccinated and infected develop robust antigen-specific IFNγ-producing CD4^+^ T (Th1) cells (6–8, 12).

Development of an adaptive CD4^+^ T-cell response requires antigen uptake, processing, and presentation of proteins by antigen-presenting cells (APCs) to the T-cell receptor (TCR) (reviewed in reference 13). Uptake can occur via phagocytosis, receptor-mediated endocytosis, or pinocytosis. Following proteolytic degradation in endolysosomal compartments, peptides ranging in size from 10 to 25 amino acids (average length ~15 amino acids) (14–16) form a complex with major histocompatibility complex class II (MHC-II) and are transported to the cell surface for presentation. Within the peptide is a 9-amino-acid core sequence, of which the first, fourth, sixth, and ninth amino acids bind to MHC-II, with the other five amino acids exposed to the TCR on the CD4^+^ T cell (17, 18). The amino acids flanking the core may also contribute to binding (18).

Despite the recognition that adaptive immunity is paramount for host defenses and vaccine-mediated protection against cryptococcosis, information is limited regarding the epitopes in cryptococcal proteins that stimulate antigen-specific CD4^+^ T-cell responses. CD4^+^ T-cell epitope identification could be useful for the development of hybrid multiepitope vaccines and diagnostic tests. Mapping stimulatory MHC-II peptide epitopes within antigenic proteins is challenging in humans because the human leukocyte antigen (HLA) MHC-II is highly polymorphic, and individuals typically have different alleles encoded by the HLA DR, DP, and DQ MHC class II loci (13, 19, 20). Immunoinformatic programs can predict the binding affinity of peptides to individual MHC-II molecules (21, 22). While useful, the programs do not account for antigen processing (such as cleavage by cathepsins and other proteases), posttranslational modifications, T-cell precursor frequency, and the T-cell-exposed motifs that bind to the TCR (13, 17, 18, 21, 23). Ultimately, experimental validation of *in silico* predictions is needed (16).

In the present study, we used overlapping peptide libraries to identify CD4^+^ T-cell epitopes present within Cda2 and Cda1 following vaccination of C57BL/6 and DR4 mice. MHC-II peptide presentation in C57BL/6 mice occurs via the IAb protein (15). DR4 mice are on the C57BL/6 background, are null for mouse MHC class II, and express the relatively common human HLA-DR allele, HLA-DRB1*04:01 (11, 24). Thus, differences in peptide epitopes that stimulate CD4^+^ T-cell responses in C57BL/6 and DR4 mice should be attributable to the disparate MHC-II molecules expressed by the two mouse strains. The experimentally validated epitopes in Cda2 and Cda1 were compared with predicted epitopes using the Immune Epitope Database (IEDB) MHC class II tool, NetMHCIIpan 4.1 BA (22). Finally, a hybrid fusion protein created using regions of Cda2 and Cda1 rich in validated MHC-II/CD4^+^ T-cell epitopes protected DR4 mice when used in a vaccine containing a Th1/Th17-inducing adjuvant.

## RESULTS

### Use of long peptides to identify MHC-II epitopes in Cda2 and Cda1 stimulating CD4^+^ T cells

**Fig 1:**
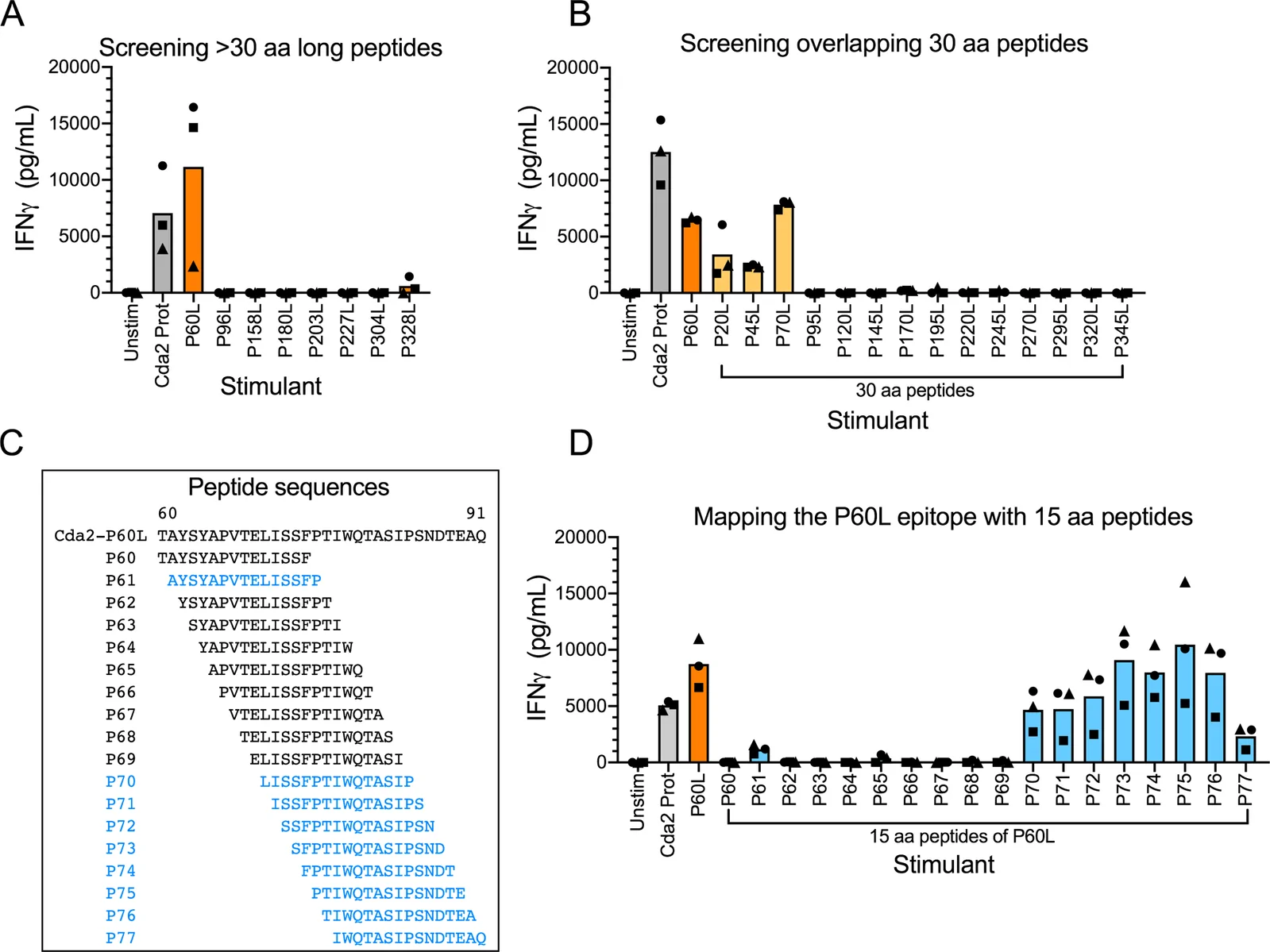
Identification and mapping of a DRB1*0401 epitope of Cda2 using long peptides (≥30 amino acids [aa]). Splenocytes from DR4 mice immunized with recombinant Cda2 protein (Cda2 Prot) were stimulated with the indicated peptides or proteins for 3 days. IFNγ was then measured in the supernatant by ELISA. Cda2 Prot was used as the positive-control stimulant, and unstimulated (Unstim) splenocytes were used as the negative control. (**A**) Identification of a DRB1*0401 epitope of Cda2 by screening with eight previously published long peptides (25). (**B**) Screening for additional DRB1*0401 epitopes of Cda2 with sequential 30 aa overlapping peptides. (**C**) Sequences of 15 aa peptides used in panel **D** to map the epitope. (**D**) Mapping the location of the epitope identified with Cda2-P60L using 15 aa peptides that span peptide Cda2-P60L. Blue type indicates peptide sequences that were stimulatory, whereas black type denotes peptides that were not stimulatory. Each symbol shape represents the results from an individual mouse.

In previous experiments, we had synthesized eight “long peptides” (31–35 amino acids in length), which were predicted to have strong binding to the MHC-II H2-IAd allele found in BALB/c mice. When formulated as glucan particle-based vaccines, five of the peptides significantly protected BALB/c mice, but not C57BL/6 mice, from an otherwise lethal pulmonary *C. neoformans* challenge (25). In initial experiments, we immunized DR4 mice with recombinant Cda2 adjuvanted with CAF01. Splenocytes from harvested spleens were prepared and incubated with the eight peptides, and IFNγ was measured in the supernatants (Fig. 1A). The nomenclature adopted for the long peptides was PXXL, with P indicating peptide, XX locating the position of the starting amino acid of the peptide in the protein sequence (counting from the N-terminus), and L designating that it is a long peptide. Thus, the 32-amino-acid peptide P60L starts at amino acid 60 and ends at amino acid 91. The amino acid sequences of the long Cda2 peptides are shown in Table S1. Of the eight peptides, P60L clearly stimulated IFNγ above background values. The positive control, recombinant Cda2 protein (Cda2 Prot), was comparably stimulatory.

The eight long peptides include the amino acid sequences from only parts of Cda2. We next took an unbiased approach to epitope identification by screening 30-amino-acid peptides (30-mers) that overlap by five amino acids and span the entire amino acid sequence of recombinant Cda2 (Fig. 1B; Table S1). IFNγ release from splenocytes obtained from immunized DR4 mice was stimulated by P20L, P45L, and P70L. The positive control, recombinant Cda2, and the previous hit, P60L, also stimulated IFNγ. To determine which amino acids within the P60L peptide were responsible for stimulating IFNγ production, we synthesized a set of 15-amino-acid (15-mer) peptides that overlapped by 14 amino acids and encompassed the entire span of P60L (Fig. 1C). The nomenclature adopted for the 15-mer peptides was PXX, with P indicating peptide, and XX denoting the position of the starting amino acid of the peptide in the protein sequence. Positive hits, as measured by splenocyte IFNγ release, were seen after stimulation of immune splenocytes with peptides P70–P77 (Fig. 1D). A very modest response was also seen with P61.

The same approach was taken with C57BL/6 mice immunized with Cda2 (Fig. S1A and B; Table S1) as had been done with the DR4 mice. IFNγ release was stimulated by P60L and P203L in Fig. S1A; and P20L, P45L, P70L, P220L, P295L, P320L, and P345L, as shown in Fig. S1B. Finally, using overlapping 30-mers spanning Cda1, we identified CD4^+^ T-cell epitopes in DR4 and C57BL/6 mice immunized with recombinant Cda1 (Fig. S1C and D; Table S2). Two peptides that stimulated IFNγ release, P320L and P345L, were discovered in Cda1-immunized DR4 mice. For the immunized C57BL/6 mice, we also found two peptides, P120L and P270L.

### Screening with 15-amino-acid overlapping peptides to identify MHC-II epitopes in Cda2 and Cda1

A more rigorous screening approach was taken to validate the hits identified with the long peptides and to identify epitopes that may have been missed. Long peptides require uptake, processing, and presentation by APCs, whereas 15-mers can directly bind in the MHC-II pocket where they can be presented to the TCR on CD4^+^ T cells (14–16). We had peptide libraries synthesized spanning Cda2 (72 peptides) and Cda1 (71 peptides) that consisted of 15-mers overlapping by 10 amino acids. DR4 and C57BL/6 mice were immunized with either Cda2 or Cda1 adjuvanted with CAF01; spleens were harvested, and splenocytes were stimulated with the peptides from the respective Cda libraries. IFNγ levels in the supernatants served as the readout for immune stimulation. When a hit was obtained, the epitope was precisely mapped using 15-mers that overlapped by 14 amino acids. For each cryptococcal protein and MHC-II allele, the IEDB was used to generate the predicted IC_50_ values for each 15-mer within the protein (22) and plotted against the position of the peptide in the protein sequence. The IC_50_ is the nM concentration of peptide required to displace a reference peptide from the MHC-II molecule (https://iedb.org/home_v3.php). The lower the IC_50_, the stronger the binding. While minimum binding thresholds have not been well established and differ for each MHC-II allele, nearly all validated MHC-II epitopes reportedly have IC_50_ values under 1,000 nM (https://discuss.iedb.org/t/selecting-thresholds-cut-offs-for-mhc-class-i-and-ii-binding-predictions/182).

**Fig 2:**
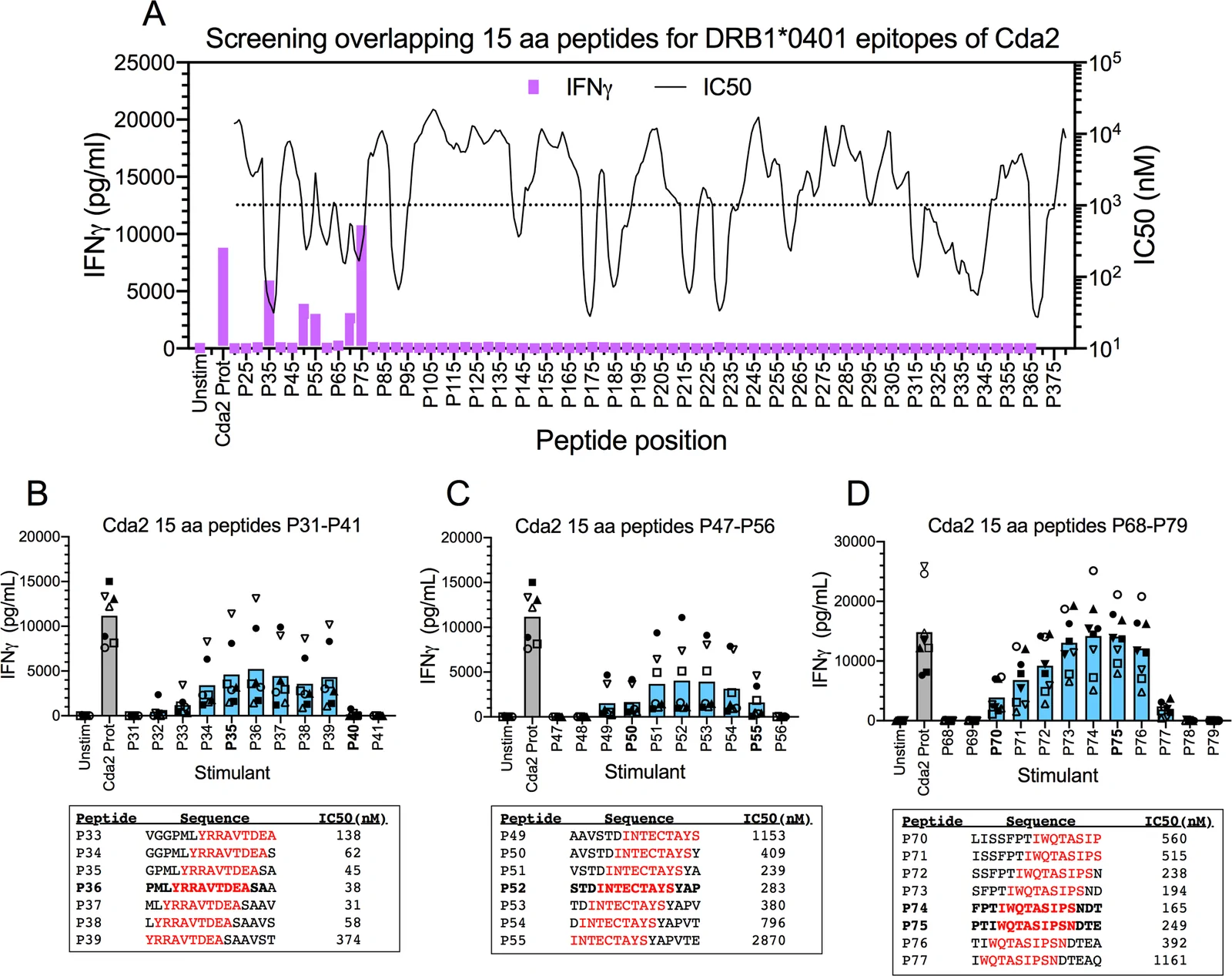
Mapping DRB1*0401 epitopes of Cda2 using 15 aa overlapping peptides. As in Fig. 1, except splenocytes from DR4 mice vaccinated with recombinant Cda2 protein (Cda2 Prot) were assayed using 15 aa peptides as stimulants. (**A**) Screening the Cda2 recombinant protein with overlapping 15 aa peptides (*n* = 3 mice). Average IFNγ generated by each stimulant is represented by the height of its respective purple bar. Predicted IC_50_ values (nM) for sequential 15 aa peptides of recombinant Cda2 protein were plotted as a solid line. The dotted horizontal line is set at 1,000 nM as most validated MHC-II epitopes have IC_50_ values under this value. (**B–D**) Three epitopes discovered in panel **A** were mapped using splenocytes from immunized mice stimulated with 15 aa peptides that overlap by 14 aa. In these graphs the P#s in bold were peptides tested as stimulants in panel A**.** Below each graph there are tables listing the sequences of peptides that stimulated IFNγ generation and their respective IC_50_ value predicted for binding to the human DRB1*0401 MHC-II allele. The shared nine aa core sequence of each epitope is in red type. The peptide with the nine aa core positioned in the middle of the peptide is in bold. Data are from two independent experiments, each with three to four mice. Each symbol shape represents results from an individual mouse. Closed symbols are from the first experiment, whereas open symbols are from the repeat experiment. Statistical comparisons are shown in Table S3A.

The DRB1*0401 epitopes identified using DR4 mice immunized with Cda2 were mapped first (Fig. 2). Figure 2A shows the IFNγ levels stimulated by each of the 15-mers from the Cda2 peptide library. The predicted IC_50_ values are superimposed on the graph. Hits were obtained for peptides P35, P50, P55, P70, and P75. Each of the 15-mer sequences is also found in a 30-mer: P35 in P20L; P50 and P55 in P45L; and P70 and P75 in P60L and P70L. Both screens validated that the DRB1*0401 MHC-II epitopes are confined to the N-terminal region of Cda2. We next mapped each of the epitopes using 15-mers that overlapped by 14 amino acids. With this mapping, we found an epitope that included P35 (Fig. 2B), one that included P50 and P55 (Fig. 2C), and a third one that included P70 and P75 (Fig. 2D). Thus, Cda2 contains three distinct epitopes that stimulate splenocytes from Cda2-immunized DR4 mice. A 9-amino-acid core sequence for each epitope was predicted by NetMHCIIpan 4.1 BA software as being shared among the epitope-defining peptides that were stimulatory. The three distinct epitopes within Cda2 were named P36, P58, and P68 based on the P# of the peptide containing the 9-amino acid sequence that elicited high IFNγ levels while having a low predicted IC_50_.

**Fig 3:**
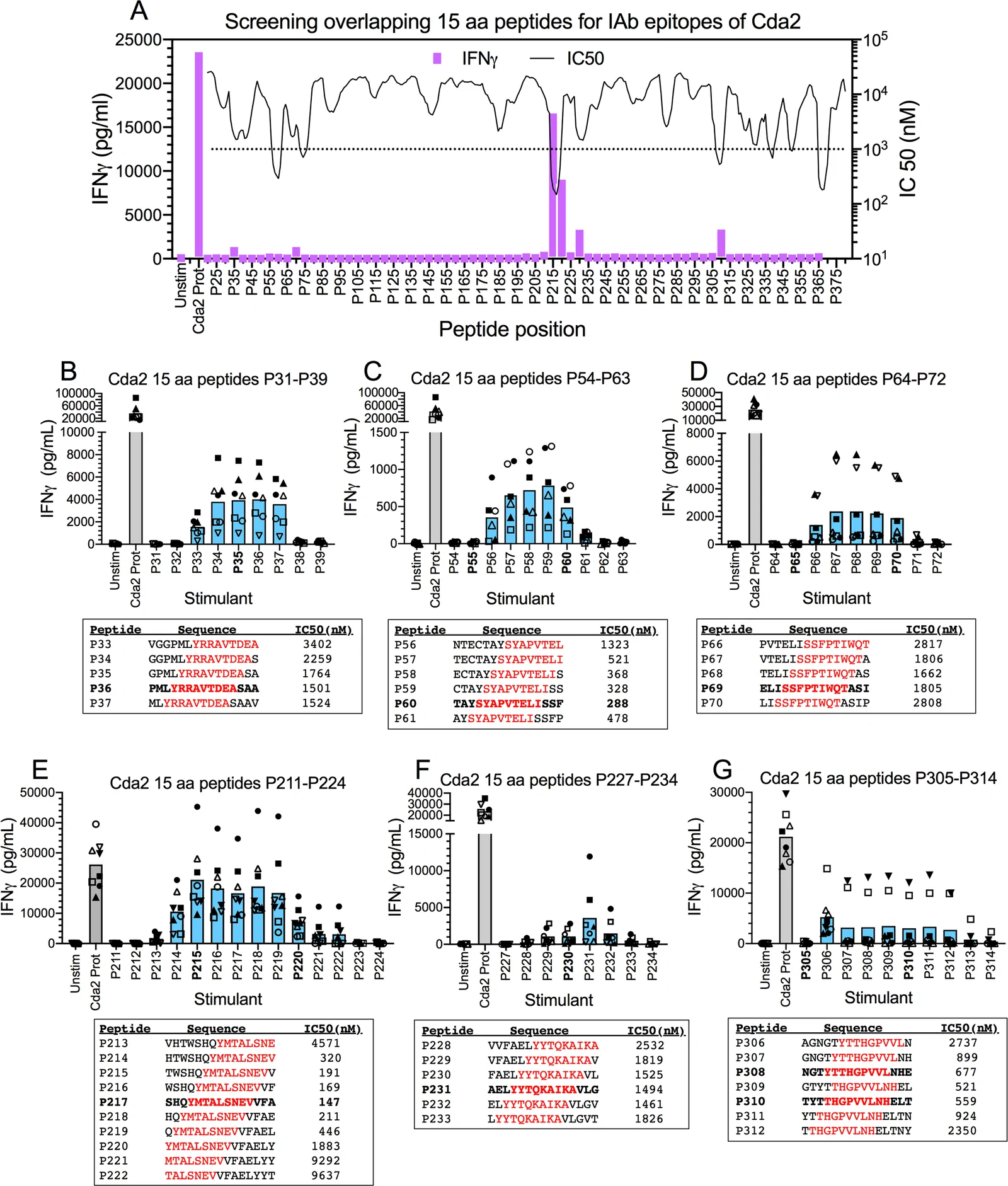
Mapping IAb epitopes of Cda2 using 15 aa overlapping peptides. As in Fig. 2, except splenocytes from C57BL/6 mice vaccinated with recombinant Cda2 protein (Cda2 Prot) were assayed. (**A**) Screening the Cda2 recombinant protein with overlapping 15 aa peptides (*n* = 3 mice). Averaged IFNγ generated by each stimulant is represented by the height of its respective purple bar. Predicted IC_50_ values (nM) for sequential 15 aa peptides of recombinant Cda2 protein were plotted as a solid line. The dotted horizontal line is set at 1,000 nM, as most validated MHC-II epitopes have IC_50_ values under this value. (**B–G**) Six epitopes were mapped using splenocytes from immunized mice stimulated with 15 aa peptides that overlap by 14 aa. In these graphs the P#s in bold were peptides tested as stimulants in panel **A**. Below each graph there are tables listing the sequences of peptides that stimulated IFNγ generation and their respective IC_50_ values predicted for binding to the mouse IAb MHC-II allele. The shared nine aa core sequence of each epitope is in red type. The peptide with the nine aa core positioned in the middle of the peptide is in bold. Data are from two independent experiments, each with three to four mice. Each symbol shape represents results from an individual mouse. Closed symbols are from the first experiment, whereas open symbols are from the repeat experiment. Statistical comparisons are shown in Table S3B.

We next mapped the IAb MHC-II epitopes in C57BL/6 mice immunized with Cda2 (Fig. 3). Although the predicted IC_50_ values revealed relatively few potential epitopes with threshold values less than 1,000 nM (Fig. 3A), the long peptides P20L, P45L, P60L, and P70L, and 15-mers P35 and P70 identified epitopes in the same region as those mapped using DR4 mice. Fortunately, we were able to repurpose the 15-mers that overlap by 14 amino acids to do the fine mapping. Three IAb MHC-II epitopes were also discovered in this region. The epitope mapped in Fig. 3B is shared with its DRB1*0401 counterpart and has the same 9-amino-acid core. The epitope in Fig. 3C is found within the long peptide P45L but was not identified in the screen with the 15-mers. The epitope in Fig. 3D shares a stimulatory peptide, P70, with the DRB1*0401 epitope shown in Fig. 2C. Three additional epitopes were identified in the screens using C57BL/6 mice and were mapped in Fig. 3E through G. The epitope in Fig. 3E was identified with long peptides P203L, P220L, and with 15-mers P215 and P220. This epitope appears to generate the dominant IFNγ response. The epitope in Fig. 3F is near the one mapped in Fig. 3E; its 15-mers are also embedded in the 30-mer peptide P220L but were clearly distinguished in the 15-mer screen by stimulation of P230. The epitope in Fig. 3G was identified in the screens by 30-mer P295 and 15-mer P310. Interestingly, of the six epitopes that were discovered and their 9-amino-acid core sequences identified (Fig. 3B through G), three of these IAb epitopes had predicted IC_50_ values above 1,000 nM.

**Fig 4:**
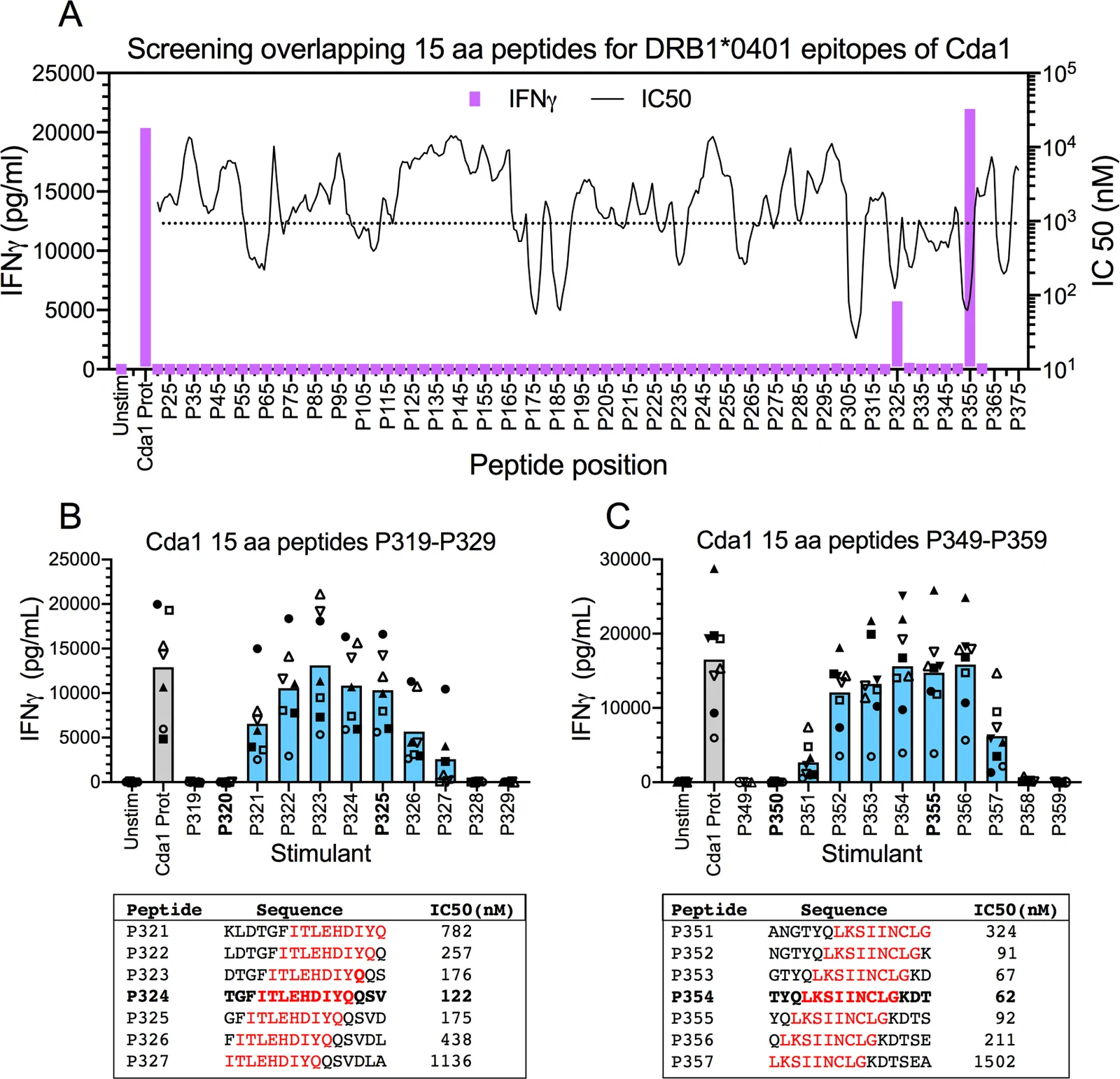
Mapping DRB1*0401 epitopes of Cda1 using 15 aa overlapping peptides. As in Fig. 2, except splenocytes from DR4 mice vaccinated with recombinant Cda1 protein (Cda1 Prot) were assayed. (**A**) Screening the Cda1 recombinant protein with overlapping 15 aa peptides (*n* = 3 mice). Averaged IFNγ generated by each stimulant is represented by the height of its respective purple bar. Predicted IC_50_ values (nM) for sequential 15 aa peptides of recombinant Cda1 protein were plotted as a solid line. The dotted horizontal line is set at 1,000 nM, as most validated MHC-II epitopes have IC_50_ values under this amount. (**B and C**) Two epitopes were mapped using splenocytes from immunized mice stimulated with 15 aa peptides that overlap by 14 aa. In these graphs the P#s in bold were peptides tested as stimulants in panel **A**. Below each graph there are tables listing the sequences of peptides that stimulated IFNγ generation and their respective IC_50_ value predicted for binding to the human DRB1*0401 MHC-II allele. The shared nine aa core sequence of each epitope is in red type. The peptide with the nine aa core positioned in the middle of the peptide is in bold. Data are from two independent experiments, each with three to four mice. Each symbol shape represents results from an individual mouse. Closed symbols are from the first experiment, whereas open symbols are from the repeat experiment. Statistical comparisons are shown in Table S4A.

**Fig 5:**
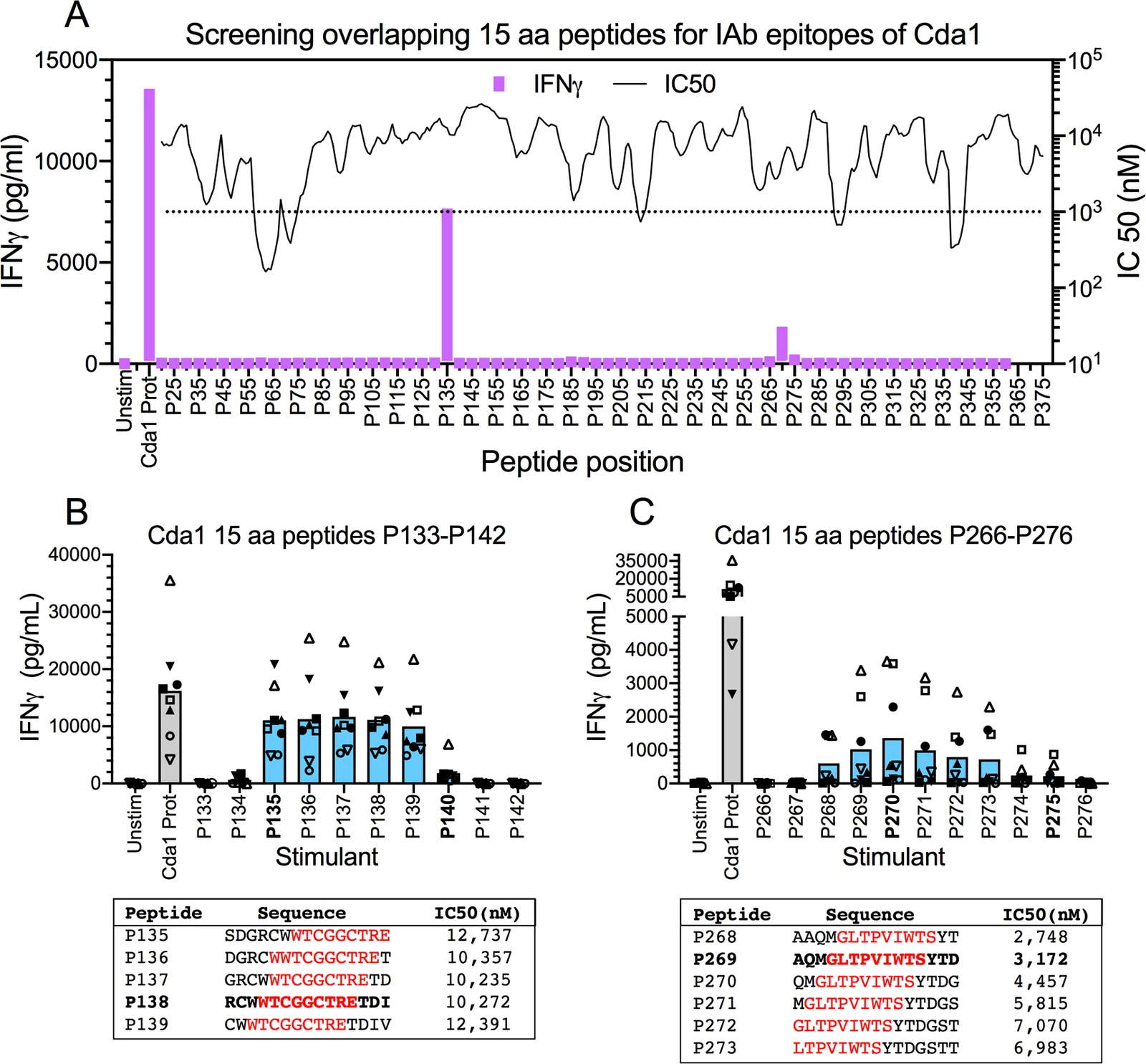
Mapping IAb epitopes of Cda1 using 15 aa overlapping peptides. As in Fig. 2, except splenocytes from C57BL/6 mice vaccinated with recombinant Cda1 protein (Cda1 Prot) were assayed using 15 aa peptides as stimulants. (**A**) Screening the Cda1 recombinant protein with overlapping 15 aa peptides (*n* = 3 mice). Averaged IFNγ generated by each stimulant is represented by the height of its respective purple bar. Predicted IC_50_ values (nM) for sequential 15 aa peptides of recombinant Cda1 protein were plotted as a solid line. The dotted horizontal line is set at 1,000 nM, as most validated MHC-II epitopes have IC_50_ values under this amount. (**B and C**) Two epitopes were mapped using splenocytes from immunized mice stimulated with 15 aa peptides that overlap by 14 aa. In these graphs the P#s in bold were peptides tested as stimulants in panel **A**. Below each graph there are tables listing the sequences of peptides that stimulated IFNγ generation and their respective IC_50_ value predicted for binding to the mouse IAb MHC-II allele. The shared nine aa core sequence of each epitope is in red type. The peptide with the nine aa core positioned in the middle of the peptide is in bold. Data are from two independent experiments, each with four mice. Each symbol shape represents results from an individual mouse. Closed symbols are from the first experiment, whereas open symbols are from the repeat experiment. Statistical comparisons are shown in Table S4B.

MHC-II epitopes in DR4 and C57BL/6 mice immunized with Cda1 were determined using the same methodology used for discovering Cda2 epitopes. Consistent with the screening performed with the long peptides, two distinct DRB1*0401 epitopes were validated in immunized DR4 mice using the overlapping 15-mers screen (Fig. 4A). The sequence of the 15-mer P325 is in the sequence of the 30-mer P320L, and the sequence of 15-mer P355 is within the 30-mer P345L. The mapping of each epitope and identification of their respective 9-amino-acid core sequences are shown in Fig. 4B and C, respectively. Two IAb epitopes were also validated using vaccinated C57BL/6 mice (Fig. 5). The sequence of the 15-mer P135 is in 30-mer P120L, and that of 15-mer P270 is in 30-mer P270L. Mapping of each epitope and identification of their respective 9-amino-acid core sequences are shown in Fig. 5B and C, respectively. The sequences of the stimulating peptides of the epitope mapped in Fig. 5B contain three cysteines, which is unusual. The predicted IC_50_ values for the DRB1*0401 epitopes were low (Fig. 4B and C). However, for the C57BL/6 mice, the IAb IC_50_ values were very high (Fig. 5B and C), even exceeding >10,000 nM for the epitope mapped in Fig. 5B.

In summary, the identification and mapping of the nine epitopes of Cda2 and four epitopes of Cda1 has shown that these MHC-II epitopes span five to ten sequential 15-amino-acid peptides that generate IFNγ above the background levels of flanking peptides. For each of the epitopes we identified, statistical analysis by ordinary one-way ANOVA was done using pairwise comparisons of unstimulated IFNγ values to those that were peptide-stimulated (Tables S3 and S4). *P* values of stimulatory peptides were lower than those for the non-stimulatory flanking peptides.

### Effect of CD4^+^ T-cell depletion on IFNγ release stimulated by validated epitopes

**Fig 6:**
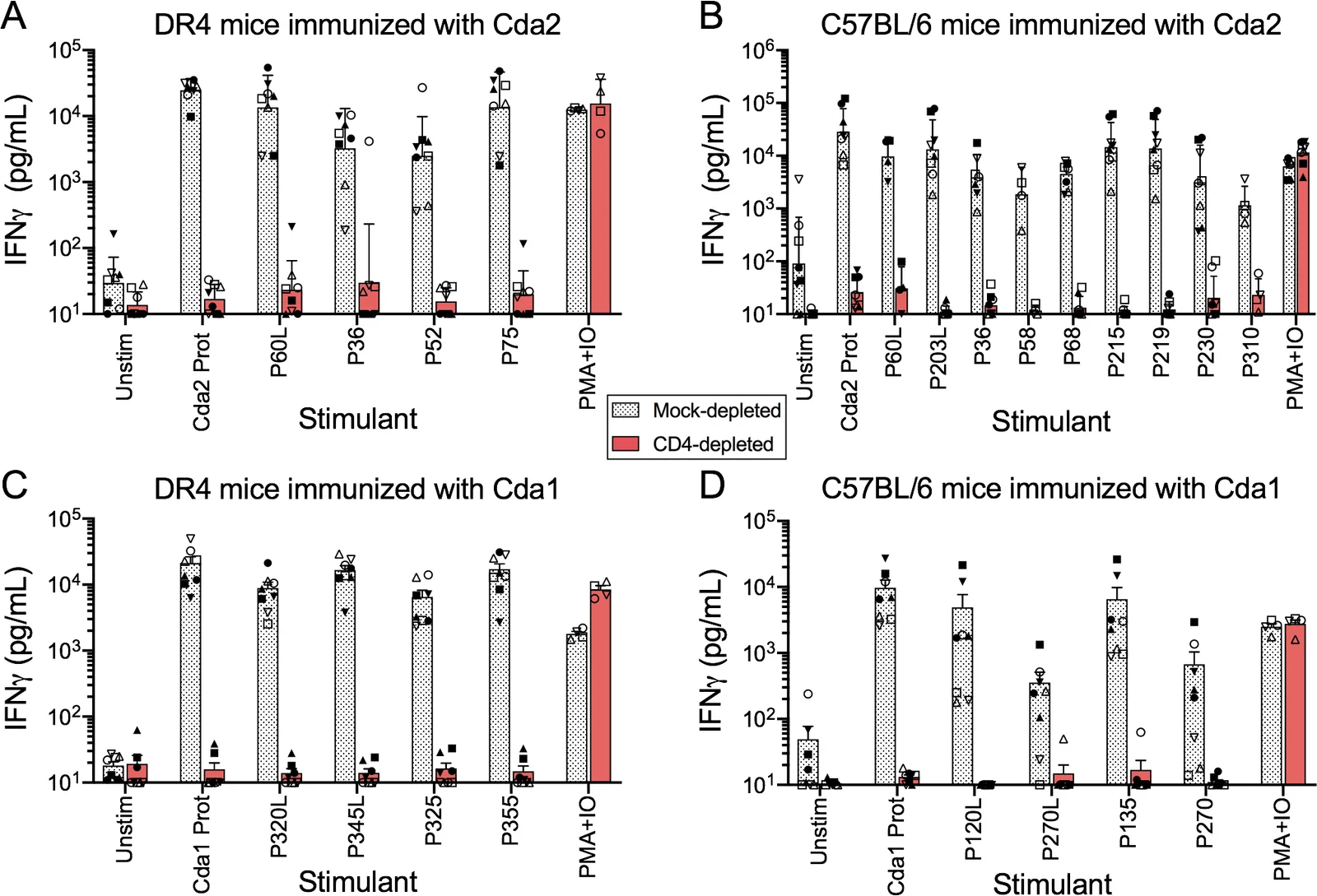
The effect of CD4^+^ T-cell depletion on splenocyte stimulation by Cda1 and Cda2 epitopes. DR4 and C57BL/6 mice were immunized with either Cda2 (**A and B**) or Cda1 (**C and D**) proteins, respectively. Spleens were harvested 2 weeks after the last vaccination, and single-cell suspensions were prepared. The splenocytes were then either mock-depleted or depleted of CD4^+^ T cells using magnetic beads (CD4-depleted). The mock-depleted group was subjected to the same conditions, except no beads were added. The two splenocyte populations were either left unstimulated (Unstim) or stimulated with the indicated protein (Prot) or peptide. Phorbol 12-myristate 13-acetate + ionomycin (PMA + IO) was used as a positive control. Cells were cultured for 3 days, following which IFNγ levels in the supernatant were measured by ELISA. Data are from two independent experiments, each with four mice. Each symbol shape represents results from an individual mouse. Closed symbols are from the first experiment, whereas open symbols are from the repeat experiment.

The adjuvant we used, CAF01, stimulates strong Th1- and Th17-biased CD4^+^ T-cell responses (8, 26). However, we have also observed weak Th1-biased CD8^+^ T-cell responses following CAF01-adjuvanted vaccination (8). Therefore, we next determined if CD4^+^ T cells were the source of the IFNγ following stimulation of splenocytes from immunized DR4 and C57BL/6 mice for validated epitopes of Cda2 and Cda1. To accomplish this, we compared IFNγ release in splenocytes depleted of CD4^+^ T cells with mock-depleted splenocytes. Efficiency of depletion was verified by flow cytometry (Fig. S2; Table S5). Unstimulated cells served as the negative control, while cells stimulated with 12-phorbol 13-myristate acetate (PMA) plus ionomycin (IO) served as the positive control. Regardless of the mouse strain and the protein used for immunization, peptide-stimulated IFNγ levels of each epitope were reduced to background concentrations when CD4^+^ T cells were depleted (Fig. 6A through D). The cells retained the capacity to make IFNγ, as the PMA + IO-stimulated levels of IFNγ were roughly the same when comparing CD4-depleted to mock-depleted splenocytes. Thus, CD4^+^ T cells are the apparent source of the epitope-stimulated IFNγ release from splenocytes of Cda2- and Cda1-immunized mice.

### Construction of a Cda2/1 hybrid protein and its testing in a vaccine model

**Fig 7:**
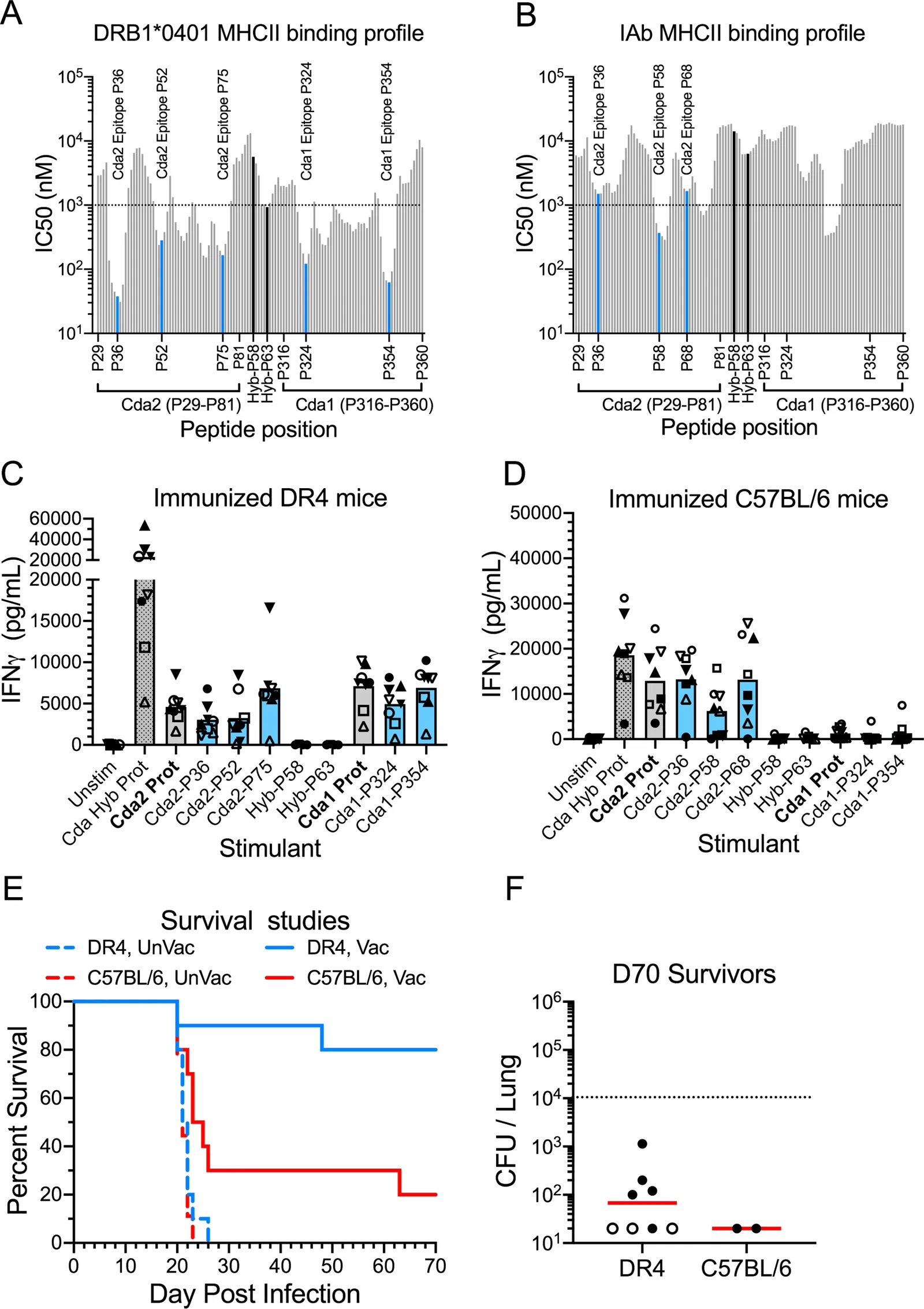
Analysis of the Cda2/1 hybrid protein as an immunogen and vaccine antigen. (**A and B**) IC_50_ profiles of the sequential 15 aa peptides that comprise the Cda2/1 protein for MHC-II alleles HLA-DRB1*0401 (DR4 mice, in panel **A**) and H2-IAb (C57BL/6 mice in panel **B**), respectively. Blue bars denote the location of 15 aa peptide sequences that stimulated IFNγ in immune splenocytes. The black bars show the location of 15 aa peptides (Hyb-P58 and Hyb-P63) containing aa sequences present in Cda2 and Cda1 (see Fig. S3). The dotted horizontal line is set at 1,000 nM, as most validated MHC-II epitopes have IC_50_ values under this amount. (**C and D**) Splenocytes from DR4 (**C**) and C57BL/6 (**D**) mice immunized with the Cda2/1 hybrid protein were stimulated with the 15 aa peptides identified with the blue and black bars in panels **A** and **B**. The negative and positive controls were unstimulated splenocytes (Unstim) and splenocytes stimulated with recombinant Cda2/1 protein (Cda Hyb Prot). After 3 days of incubation, IFNγ was measured by ELISA in supernatants of splenocyte cultures. Data are from two independent experiments, each with four mice. Each symbol shape represents results from an individual mouse. Closed symbols are from the first experiment, whereas open symbols are from the repeat experiment. (**E and F**) DR4 and C57BL/6 mice were left unvaccinated (UnVac) or vaccinated (Vac) with Cda2/1 and then given a pulmonary challenge with *C. neoformans*. Survival curves are shown in panel **E** and represent data from two independent experiments, each with five mice per group. *P* < 0.01 comparing survival curves of vaccinated DR4 and C57BL/6 mice. *P* < 0.0001 comparing UnVac and Vac mice for both DR4 and C57BL/6 mice. Lung CFUs of mice that survived to D70 are shown in panel **F**. Closed and open circles represent CFUs of surviving mice from the first and second experiments, respectively. The dotted horizontal line at 10^4^ CFUs denotes the challenge *C. neoformans* inoculum.

For DR4 mice, the three validated epitopes (P36, P52, and P75, shown in Fig. 2) in Cda2 are near the N-terminus, while both validated epitopes of Cda1 (P324 and P354, shown in Fig. 4) are near the C-terminus (Fig. 2 and 4). We next constructed a hybrid protein in *Escherichia coli*, which we named Cda2/1, by combining amino acids 29–95 of Cda2 with amino acids 316–374 of Cda1 (Fig. S3). The DRB1*0401 and IAb binding profiles, based on predictions using the IEDB, are shown in Fig. 7A and B, respectively. Next, splenocyte preparations were made from DR4 mice immunized with Cda2/1 and then incubated with P36, P52, P75, P324, and P354. Each of the five peptides stimulated IFNγ release, as did full-length Cda2, Cda1, and the Cda2/1 hybrid protein (Fig. 7C). IFNγ secretion was not seen following stimulation with two peptides (designated Hyb-P58 and Hyb-P63), which contained amino acid sequences from both Cda2 and Cda1.

For the C57BL/6 mice, only the P36, P58, and P68 IAb epitopes of Cda2 (mapped in Fig. 3A through C) were retained in Cda2/1. The three other validated IAb epitopes in Cda2 and the two validated IAb epitopes of Cda1 were not included in the Cda2/1 construct. As expected, P36, P58, and P68 stimulated IFNγ production in splenocytes obtained from C57BL/6 mice immunized with Cda2/1 (Fig. 7D). Peptides Hyb-P58 and Hyb-P63 and peptides containing sequences from the Cda1 portions of Cda2/1 did not stimulate. When the recombinant proteins were used as stimuli, the Cda2 and hybrid Cda2/1 proteins, but not the Cda1 protein, induced IFNγ production. This result was consistent with the absence of Cda1 IAb epitopes in the Cda2/1 construct.

In the final set of experiments, we vaccinated DR4 and C57BL/6 mice with Cda2/1 adjuvanted with CAF01. Mice received a prime vaccine followed 3 weeks later by a booster dose. Both mouse strains were protected from *C. neoformans* challenge by the Cda2/1 vaccine, with protection significantly greater in the DR4 mice (Fig. 7E). Many of the surviving mice had undetectable lung CFUs (Fig. 7F).

## DISCUSSION

Multiple lines of evidence support the inclusion of *C. neoformans* Cda2 and Cda1 in subunit cryptococcal vaccine formulations. Cda2 and Cda1 protein sequences lack human homologs, are abundantly expressed during cryptococcal infection, are highly conserved among cryptococcal species, and elicit protective and immunodominant Th1-biased CD4^+^ T-cell responses in murine models of cryptococcosis (6–11, 27, 28). However, to be effective, CD4^+^ T-cell vaccines need to account for the extensive variation in MHC-II molecules expressed in the human population (16). Here, we used experimental data, supported in part by *in silico* predictions, to map the MHC-II epitopes in Cda2 and Cda1 that stimulate CD4^+^ T-cell responses in C57BL/6 and DR4 mice. We then designed a hybrid protein containing validated epitopes of Cda2 and Cda1 to demonstrate that an adjuvanted vaccine containing the hybrid protein could protect humanized mice against an otherwise lethal cryptococcal infection.

The lower the predicted IC_50_, the tighter the predicted binding of a peptide to an MHC-II molecule, with a threshold of an IC_50_ value of <1,000 nM recommended to predict a good binder (21, 29). Using this cutoff, most 15-mer peptides present in Cda2 and Cda1 that were predicted by the IEDB to be good binders to DRB1*04:01 nevertheless did not stimulate IFNγ responses in splenocytes from immunized mice. An antigen-stimulated CD4^+^ T-cell response requires antigen processing and presentation of the peptide to the TCR. Even assuming the accuracy of the bioinformatic predictions, loss of epitopes can occur if antigen processing does not lead to MHC-II presentation of the peptide. This could occur during processing by cathepsins or if posttranslational modifications, such as glycosylation, interfere with presentation (23). Moreover, even if peptide binding to MHC-II is strong, the precursor frequency of TCRs capable of binding to the T-cell-exposed motif on the peptide could be insufficient to initiate a CD4^+^ T-cell response (30). Regardless of the reasons why a predicted MHC-II binding epitope is not validated, these data emphasize the importance of verifying *in silico* predictions experimentally.

Unexpectedly, we also validated epitopes that were not predicted to bind well to MHC-II. For example, the Cda1 P135 peptide stimulated robust IFNγ production in splenocytes from immunized C57BL/6 mice despite having a predicted IC_50_ values of >10,000 nM to H2-IAb. This epitope would not have been discovered had we not taken an unbiased approach to epitope discovery by screening libraries of overlapping peptides rather than just examining peptides with predicted IC_50_ values of <1,000 nM. The possibility that the IFNγ was from cells other than CD4^+^ T cells was effectively eliminated, as IFNγ production was reduced to background levels after *ex vivo* depletion of CD4^+^ T cells from splenocytes. It is important to note, however, that our experimental setup, including the use of CAF01 as an adjuvant and screening with 15-mer peptides, was designed to detect MHC-II epitopes. Immunization with an adjuvant that stimulates strong CD8^+^ T-cell responses and then screening with 9-mer peptides could validate which sequences are MHC-I epitopes.

Not surprisingly, considering the evolutionary distance between mice and humans, most peptides that were validated epitopes in DR4 mice were not stimulatory in C57BL/6 mice. About 10% of humans express at least one DRB1*0401 allele (20). The highly polymorphic nature of human MHC-II (13, 19, 20) raises the question of whether epitopes that bind to DRB1*0401 and stimulate subsequent CD4^+^ T-cell responses will do the same with other DRB1 molecules. While this needs to be determined experimentally, IEDB predictions suggest that the epitopes we validated in the DR4 mice will bind to other (although not all) DRB1 molecules (Table S6). Ultimately, to be protective, we postulate that CD4^+^ T-cell vaccines will need to account for the extensive diversity of human MHC-II alleles by containing multiple stimulatory epitopes. Experiments examining transgenic mice expressing humanized DRB1 molecules other than DRB1*0401 are planned. Contributions of other MHC-II molecules also need to be considered, given studies suggesting HLA MHC-II DQB and DPB genotypes are also associated with risk for human cryptococcosis (31, 32). Finally, our data emphasize the importance of taking into account MHC-II differences between mice and humans when developing T-cell vaccines using murine models.

We initially tested peptide libraries consisting of overlapping long peptides, ≥30 amino acids in length, but then switched to overlapping 15-mers. Compared with 15-mers, screening 30-mers requires fewer peptides given the longer length of the peptide. However, for positive hits, the mapping of the epitope to identify the core 9-amino-acid sequence that binds to MHC-II and is exposed to T cells (17, 18) may require approximately twice the number of peptides when the primary screen is with long peptides. Additionally, as noted above, 15-mers can directly bind in the MHC-II pocket, bypassing the need for antigen uptake and processing by APCs (14–16). Perhaps most importantly, when screening overlapping peptides, sequential peptides should have sufficient overlap so that epitopes are not missed. For the 15-mers, we chose an overlap of 10 amino acids, which should be sufficient for detecting nearly all epitopes, given that we experimentally showed that an epitope is defined by five to ten stimulatory 15-mers and the 9-amino-acid core region is shared by the stimulatory peptides (17, 18).

Variations in reactivity (as measured by peptide-stimulated splenocyte IFNγ production) among individual mice used as experimental replicates were observed. Generation of T-cell receptors occurs via stochastic processes, which can result in large differences in individual TCR repertoires even in inbred mouse strains (21). The relative contribution of TCR precursor frequencies to these variations seen in our mouse experiments is speculative, though. The translational implication is that cryptococcal T-cell vaccines need to contain multiple epitopes to not only account for population diversity in HLA alleles but also for individual variations in TCR precursor frequencies.

Epitope mapping has translational relevance as it informs construction of multi-epitope hybrid proteins that can be used in subunit T-cell vaccines and possibly diagnostic testing. From a manufacturing standpoint, a single protein vaccine is less expensive to manufacture and test than one containing multiple proteins. In addition, eliminating portions of the proteins that are not contributing to protection decreases the potential for eliciting autoimmunity. As a proof of principle, we constructed a relatively small (133-amino-acid) hybrid protein incorporating the epitopes in Cda2 and Cda1 that stimulated CD4^+^ T-cell responses in DR4 mice immunized with these two proteins. DR4 mice vaccinated with the hybrid protein adjuvanted with CAF01 were robustly protected against an otherwise lethal cryptococcal pulmonary challenge. In contrast, C57BL/6 mice were only minimally protected, which was not surprising as the hybrid vaccine protein included only three of the six identified Cda2 epitopes and none of the Cda1 CD4^+^ T-cell epitopes for the MHC-II of that mouse strain. Nevertheless, the hybrid protein still elicited strong *ex vivo* splenocyte IFNγ responses in immunized C57BL/6 mice; this response likely accounted for the protection that was observed.

A caveat to using hybrid proteins in vaccines is they can create neoantigens (also known as junctional epitopes) at fusion sites. In future studies, this potential pitfall may be mitigated by adding linkers or employing bioinformatics to eliminate neoantigens with undesirable properties such as possible human homology (33, 34). In addition to Cda2 and Cda1, we have discovered other proteins that, when formulated as adjuvanted vaccines, protect mice against otherwise lethal cryptococcal infections (10, 11). Epitope mapping of these proteins is underway. Once completed, we plan to create hybrid vaccines that contain multiple epitopes that elicit strong IFNγ responses. The protective efficacy of such vaccines will be tested using clinical strains representative of the global lineages of *C. neoformans* and *C. gattii* (7, 35), and in strains of mice transgenic for human MHC-II. Ultimately, though, vaccines will need to advance from preclinical studies to human testing, including in the immunocompromised populations most in need of a cryptococcal vaccine.

## MATERIALS AND METHODS

### Mice

Two strains of mice were used: C57BL/6 (Strain 000664; The Jackson Laboratory, Bar Harbor, ME, USA) and DR4 (Strain 4149; Taconic Biosciences, Rensselaer, NY, USA). Mice were maintained at the University of Massachusetts Chan Medical School (UMCMS) and housed in a pathogen-free facility.

### Immunizations and vaccinations

*C. neoformans* proteins Cda2 (CNAG_01230) and Cda1 (CNAG_05799) were cloned into plasmid pET19b to position a 10× His tag at the N-terminus of each protein (11). The DNA sequence encoded amino acids 20–378 of Cda2 and amino acids 20–374 of Cda1. The first 19 amino acids were not included as they contain the cleaved signal sequences of the two proteins. The Cda2/1 hybrid protein was generated by coupling DNA encoding amino acids 29–95 of Cda2 to amino acids 316–374 of Cda1. The DNA sequence encoding the hybrid protein was synthesized by Genscript (Piscataway, NJ, USA) and cloned into vector pET21b to include a 6× His tag at the C-terminus. Plasmids were transformed into *E. coli* strain BL21 DE3.

Recombinant proteins were expressed in *E. coli* strain BL21 DE3 using Overnight Express Instant TB medium, 1% glycerol (EMD Millipore, Burlington, MA, USA); cultures were shaken at 30°C for 18–24 h. Extraction of protein from *E. coli* cell pellets and purification of recombinant protein on His-Bind Resin (EMD Millipore) were done in buffers containing 6 M urea, as previously described (11). Stocks of recombinant protein at 5 mg/mL in 6 M urea, 20 mM Tris, pH 7.9, were stored at −80°C. Each dose of vaccine (100 μL) was formulated to contain 5 μg of protein diluted in Tris buffer with the liposomal-based CAF01 (6–8) as the adjuvant. CAF01 (Statens Serum Institut, Copenhagen, Denmark) contains 2,500 μg/mL N,N′-dimethyl-N,N′-dioctadecylammonium and 500 μg/mL α,α′-trehalose 6,6′-dibehenate in Tris buffer (10 mM, pH 7.0) supplemented with 2% glycerol. For immunization of the mice, two 100-μL subcutaneous injections were done, one injection into each side of the abdomen, and spaced about 1 cm apart. The second immunization was given 3 weeks later. Mice were euthanized 2 weeks after the second immunization and spleens were harvested.

Vaccine efficacy was determined as in our previous studies (7, 8). Briefly, groups of five mice were left unvaccinated or vaccinated as above. Two weeks after the second vaccination, mice were anesthetized with 2% isoflurane (Pivetalvet) and challenged with *C. neoformans* strain KN99α (36) by orotracheal infusion of 1 × 10^4^ CFU into the lungs. Following infection, the mice were monitored daily. Mice that survived 70 days post-infection (DPI) were euthanized. Lungs were homogenized in 4 mL phosphate-buffered saline (PBS) with 200 U/mL penicillin and 200 μg/mL streptomycin and plated on Sabouraud dextrose agar. CFUs were counted following incubation at 30°C. Detection limits were 20 CFU/lung.

### Peptides

Peptides were synthesized by Genscript. Long peptides 30–35 amino acids in length exceeded 80% purity and 15-amino-acid peptides exceeded 70% purity. Peptides were provided as lyophilized powder in vials containing either 2.0 or 0.5 mg of peptide. Each peptide was dissolved with 100% dimethyl sulfoxide (#BP231-100, Fisher Scientific) to generate stock solutions at 5 mg/mL, which were stored at −20°C.

### Splenocyte stimulation and IFNγ ELISA

Splenocytes were stimulated with proteins and peptides as described (6, 7). Briefly, spleens were homogenized in complete media (RPMI 1640 supplemented with 10% FBS, 1% HEPES, 1% GlutaMAX, and 1% penicillin–streptomycin; reagents purchased from Thermo Fisher Scientific) using the piston of a 3-mL syringe on a 70-µm cell strainer. The cell pellet was collected following centrifugation, red blood cells were lysed, and the splenocytes were washed twice in complete media. The concentration and viability of cells were determined with Trypan blue (Bio-Rad Laboratories, Hercules, CA, USA) using a T20 cell counter (Bio-Rad). Splenocytes were plated in tissue culture-treated 96-well round bottom plates (#3799, Corning Inc, Corning, NY, USA) at 1 × 10^6^ cells/well. Cells were left unstimulated or stimulated with recombinant protein (5 µg/mL) or peptide (2.5 µg/mL). Duplicate wells were made for each stimulant. Incubations were at 37°C in humidified air supplemented with 5% CO_2_. Splenocytes were cultured for 3 days in complete media. Plates were then centrifuged, supernatants were collected and stored at −80°C. IFNγ concentrations in thawed supernatants were determined with the R&D Systems Mouse IFNγ DuoSet ELISA Kit (Bio-Techne, Minneapolis, MN, USA) according to the manufacturer’s instructions. Samples were diluted 1:2, or if needed 1:10. The lowest standard was 10 pg/mL. Values <10 pg/mL were arbitrarily assigned a concentration of 5 pg/mL.

### CD4^+^ T-cell depletion

Single-cell splenocyte suspensions, obtained as described above, were divided into two groups: mock-depleted and CD4-depleted cells. For the CD4-depleted group, splenocytes were depleted of CD4^+^ T cells using the Mouse CD4 (L3T4) MicroBeads (#130-117-043, Miltenyi Biotec, Bergisch Gladbach, Germany), a MACS LD column (#130-042-901, Miltenyi Biotec), and a QuadroMACS separator following the manufacturer’s instructions. Briefly, splenocytes (1 × 10^8^ cells/mL in MACS Separation buffer (#130-091-221, Miltenyi Biotec) were mixed with CD4 (L3T4) MicroBeads (10% [vol/vol]) and incubated for 10 min at 4°C. MACS LD columns were placed in the magnetic field of the QuadroMACS separator and rinsed with 2 mL of separation buffer. To remove clumps, cell suspensions were passed through 30 µm pre-separation filters and then transferred onto the pre-rinsed LD column, followed by two subsequent column washes with 1 mL of separation buffer. The unbound (CD4-depleted) cells were collected in the flow-through solution. The mock-depleted group was submitted to the same conditions, except only buffer was used with no beads added. Splenocytes in the two groups of cells were then left unstimulated or stimulated with the respective recombinant protein (5 µg/mL) or peptide (2.5 µg/mL). Phorbol 12-myristate 13-acetate (PMA) plus ionomycin (IO) at 10 ng/mL and 1 μg/mL, respectively, served as a positive control. Cells were cultured for 3 days at 37°C with 5% CO_2_, stored at −80°C, and IFNγ levels in the culture supernatant were measured by ELISA.

### Flow cytometry

Bovine serum albumin (BSA) was added to 1× PBS at a concentration of 0.5% as FACS buffer for flow cytometry staining. Splenocytes from total (sham-depleted) or CD4-depleted cells, at 1 × 10^6^ cells per sample, were washed with FACS buffer and stained for 30 min with LIVE/DEAD Fixable Green dead cell stain kit (#L34970, Invitrogen by Thermo Fisher Scientific) according to the company’s instructions. Next, cells were washed and subsequently stained for 30 min in the dark at 4°C with the anti-mouse cell surface markers CD45-APC/cyanine7 (clone: QA17A26), CD3-PE (clone: 145-2C11), CD4-PerCP/cyanine5.5 (clone: GK1.5), and CD8-APC (clone: 53-6.7). All conjugated antibodies were from BioLegend (San Diego, CA, USA) and used for staining at a dilution of 1:200. After staining, cells were washed, fixed with 2% paraformaldehyde (PFA), and acquired with a 5-laser Bio-Rad ZE5 Cell Analyzer flow cytometer (Bio-Rad). Data analysis was performed with FlowJo Software, version 10.8 (BD, Franklin Lakes, NJ, USA), and gating was established using FMO controls.

### Data analysis

GraphPad Prism, version 10.1.2 (GraphPad Software, La Jolla, CA, USA) was used for graphing the data and statistical analysis. The IEDB website (www.IEDB.org) was the source for tools to predict IC_50_ values for peptides sequentially spanning the Cda2 and Cda1 protein sequences. NetMHCIIpan 4.1 BA was used to assign IC_50_ values to each 15-amino-acid peptide and deduce 9-amino-acid core sequences for MHC-II epitopes. One-way analysis of variance (ANOVA) was used to compare splenocyte IFNγ responses following stimulation with 15 aa peptides. Kaplan-Meier survival curves were compared using the Mantel-Cox log-rank test; *P* values were based on pairwise comparisons.

## Data Availability

The authors will make all data fully available and without restriction. Epitope information has been uploaded to the IEDB (www.iedb.org). The Cda2 Reference IDs in IEDB are 1040799, 1040334, 1047809, 1048034, and 1049025. The Cda1 Reference IDs in IEDB are 1040804, 1041101, and 1047550.

## AFTER EPUB

*[This article was published on 14 October 2026 with errors in Fig. 3. The figure was corrected in the current version, posted on 3 November 2026.]*

## SUPPLEMENTAL MATERIAL

Supplemental figures and tables mbio.02176-26-s0001.pdf Figures S1 to S3; Tables S1 to S6.

ASM does not own the copyrights to Supplemental Material that may be linked to, or accessed through, an article. The authors have granted ASM a non-exclusive, world-wide license to publish the Supplemental Material files. Please contact the corresponding author directly for reuse.
