# Supplementary material for: A protective hybrid protein vaccine informed by mapping major histocompatibility class II epitopes in Cryptococcus neoformans chitin deacetylases that stimulate CD4+ T-cell responses
Source: mBio. 2026 Oct 14;17(11):e02176-26. doi: 10.1128/mbio.02176-26 (PMC13753820; doi:10.1128/mbio.02176-26)
Supplement: Supplementary file 1 [file mbio.02176-26-s0001.pdf]

Figure S1

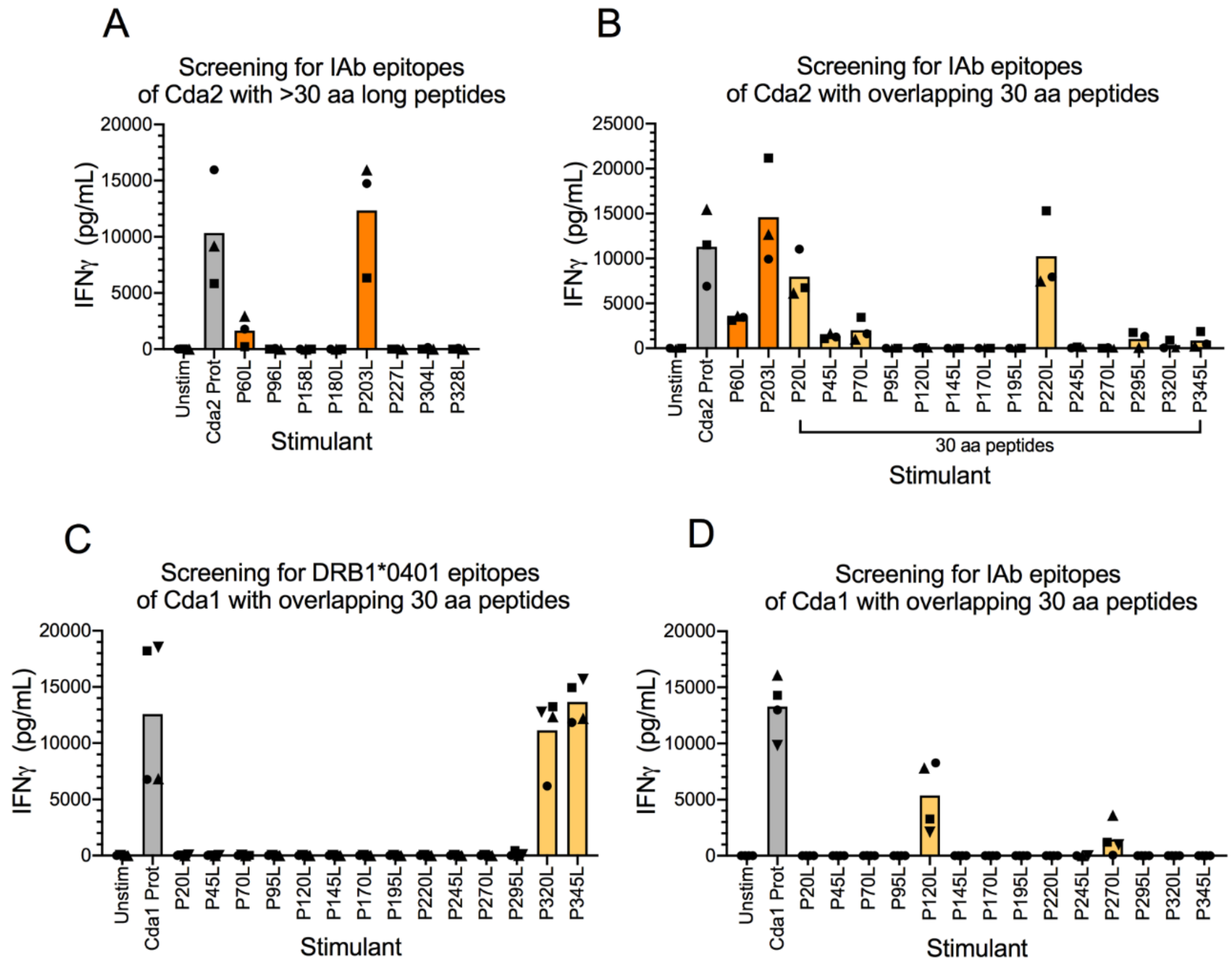

Figure S1. **Identification of IAb epitopes of Cda2, and DRB1*0401 and IAb epitopes of Cda1 using long peptides (≥30 aa)**. Methods are as in Figure 1. IAb and DRB1*0401 epitopes were identified using immunized C57BL/6 and DR4 mice, respectively. (**A**) Identification of IAb epitopes of Cda2 by screening with eight previously published long peptides[1]. (**B**) Screening for additional IAb epitopes of Cda2 with sequential 30 aa overlapping peptides in C57BL/6 mice. (**C**) Screening for DRB1*0401 epitopes of Cda1 with sequential 30 aa overlapping peptides in DR4 mice. (**D**) Screening for IAb epitopes of Cda1 with sequential 30 aa overlapping peptides in C57BL/6 mice. Each symbol shape represents results from an individual mouse.

1. Specht, C. A. *et al.* Protection of mice against experimental cryptococcosis by synthesized peptides delivered in glucan particles. *mBio* **13**, e033674 (2021).

Figure S2

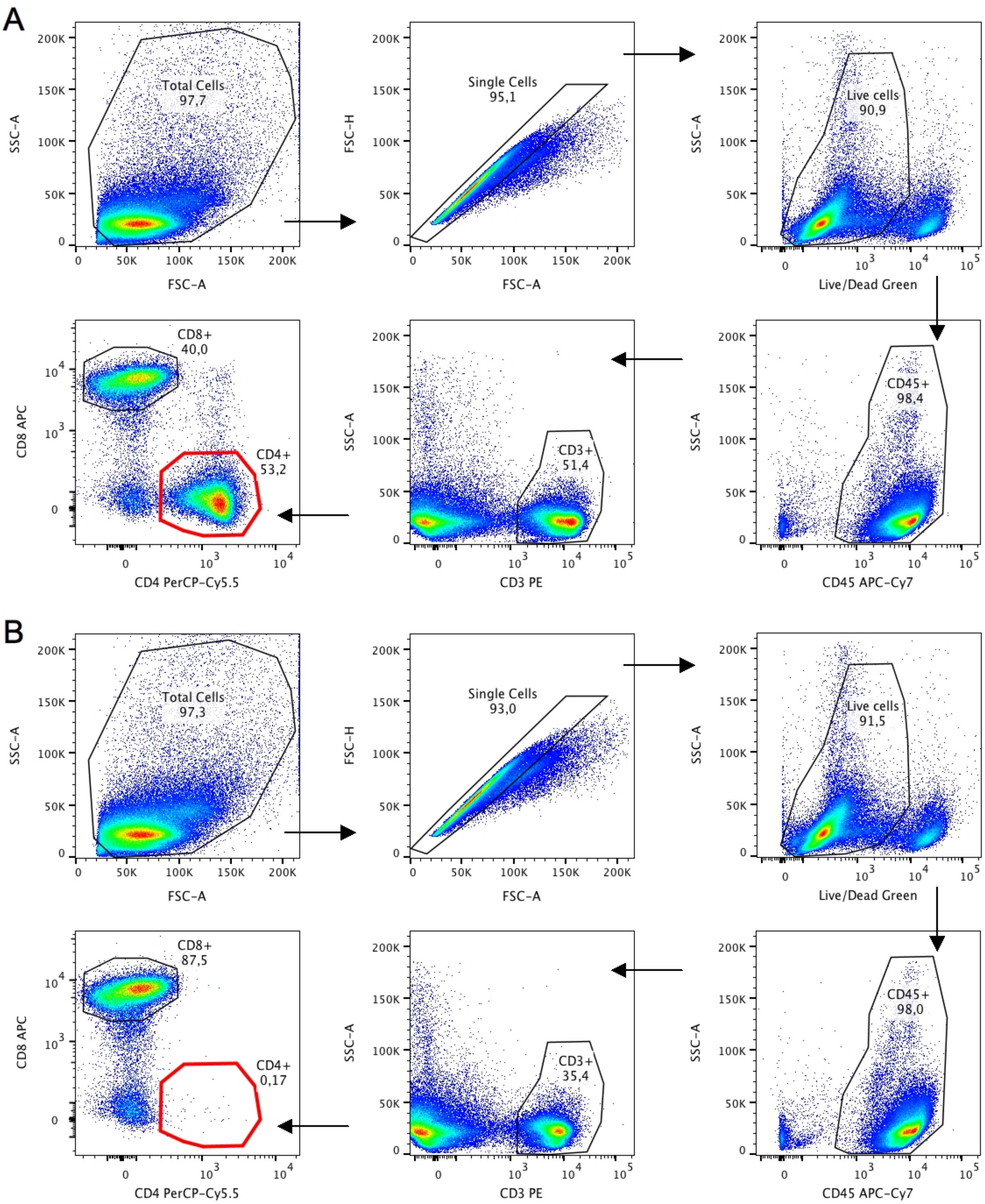

Figure S2. Flow cytometry gating strategy. Representative plots display the gating strategy used to analyze the efficiency of $CD4^+$ T cell depletion following passage over a magnetic bead

column. The examples shown are from splenocytes obtained from a C57BL/6 mouse after vaccination with Cda1 protein. (**A**) Mock-depleted. (**B**) CD4^{+} T cell-depleted. The percentages of CD4^{+} and CD8^{+} T cells were determined after sequential gating on total cells, singlets, live cells, CD45^{+} cells, and CD3^{+} T cells. Total cells were identified by excluding debris using side scatter area (SSC-A) and forward scatter area (FSC-A) plots. Singlets were then selected from total cells based on FSC-A and forward scatter height (FSC-H). Live cells were further determined by excluding dead cells based on the negative population in the Live/Dead Green channel. Next, leukocytes were defined by gating on the CD45^{+} population. The T cell population was identified by gating on CD3^{+} cells. Finally, T cell subsets were distinguished by gating on CD4^{+} and CD8^{+} cells.

Figure S3

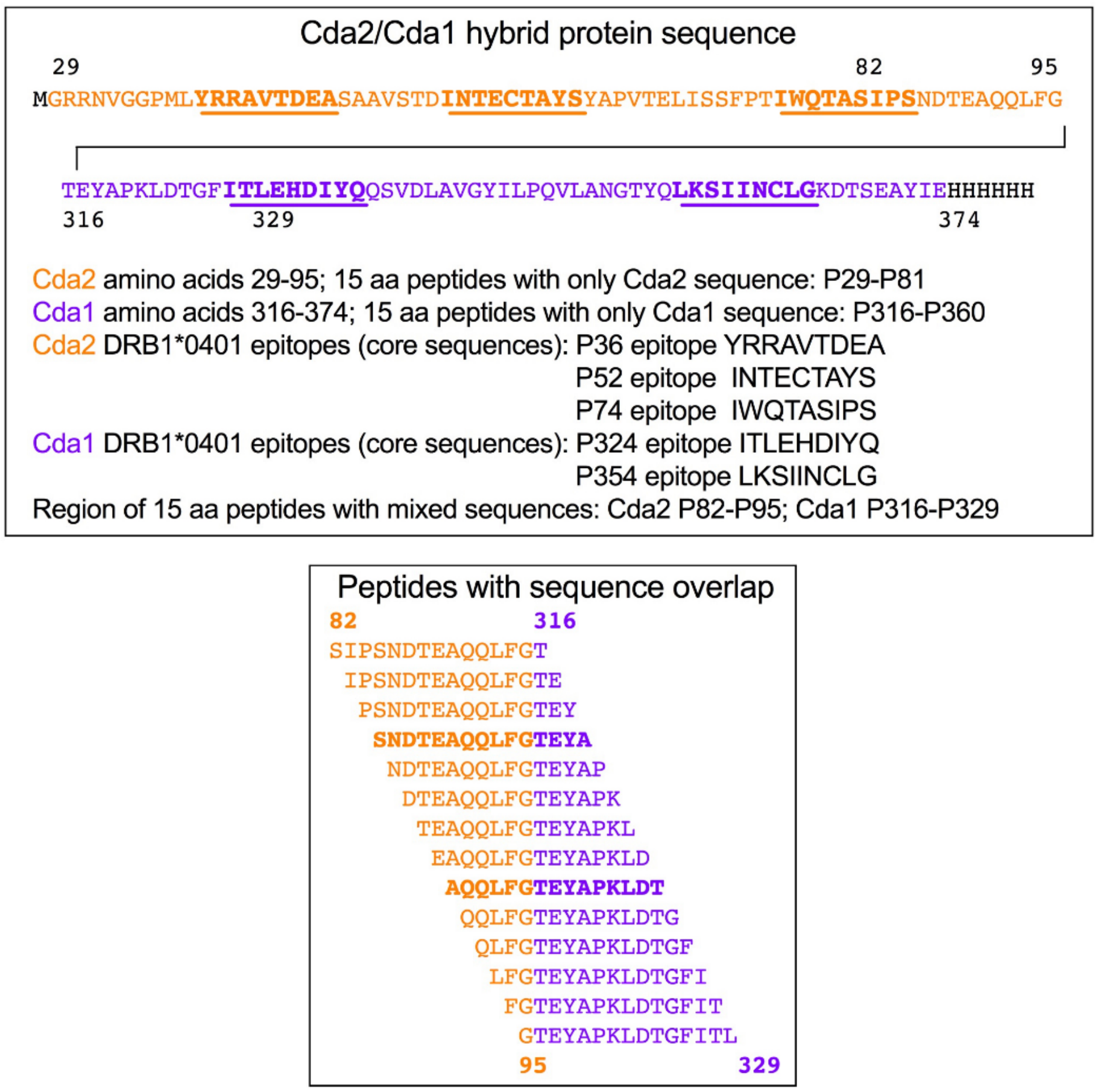

Figure S3. **Sequence of the Cda2/1 hybrid protein**. **Top.** The Cda2/1 protein combines amino acids (aa) 29-95 of Cda2 (in orange) with aa 316-374 of Cda1 (in purple). The 9 aa core sequences from validated DRB1*0401 epitopes in Cda2 and Cda1 are shown in bold and underlined. **Bottom. 15 aa sequences in Cda2/1 that contain portions of Cda2 and Cda1.** The sequences of the two 15 aa peptides tested in Figure 7, Hyb-P58 and Hyb-P63, are SNDTEAQQLFGTEYA and AQQLFGTEYAPKLDT, respectively, and shown in bold.

Table S1

>30 aa Peptide	Sequence	DR4 IFNγ (pg/mL)	C57BL/6 IFNγ (pg/mL)
**Cda2-P60L**	TAYSYAPVTELISSFPTIWQTASIPSNDTEAQ	**6,483**	**1,657**
Cda2-P96L	KINSTLNTKIPNDVPHGTPTGDWTGVNYSNSDPDC	5	23
Cda2-P158L	TWGLGFDDGPNCSHNALYDLLLENNQKATMFF	5	5
Cda2-P180L	ENNQKATMFFIGSNVLDWPLQAMRAHDEGHE	6	5
**Cda2-P203L**	RAHDEGHEICVHTWSHQYMTALSNEVVFAELY	5	**12,344**
Cda2-P227L	EVVFAELYYTQKAIKAVLGVTPQCWRPPYGDVDN	5	11
Cda2-P304L	DKAGNGTYTTHGPVVLNHELTNYTMSVFMTMFPKI	5	51
Cda2-P328L	MSVFMTMFPKIKSAFNYIVPICTAYNITQPYAES	5	28
**30 aa Peptide**	**Sequence**	**DR4 IFNγ (pg/mL)**	**C57BL/6 IFNγ (pg/mL)**
**Cda2-P20L**	HTGCGGHEIGRRNVGGPMLYRRAVTDEASA	**3,430**	**7,979**
**Cda2-P45L**	DEASAAVSTDINTECTAYSYAPVTELISSF	**2,381**	**1,329**
**Cda2-P70L**	LISSFPTIWQTASIPSNDTEAQQLFGKINS	**7,842**	**2,020**
Cda2-P95L	GKINSTLNTKIPNDVPHGTPTGDWTGVNYS	13	11
Cda2-P120L	GVNYSNSDPDCWWTHNKCTTPSNDTGLQAD	17	28
Cda2-P145L	GLQADISIAPEPMTWGLGFDDGPNCSHNAL	7	10
Cda2-P170L	SHNALYDLLLENNQKATMFFIGSNVLDWPL	224	47
Cda2-P195L	LDWPLQAMRAHDEGHEICVHTWSHQYMTAL	168	6
**Cda2-P220L**	YMTALSNEVVFAELYYTQKAIKAVLGVTPQ	57	**10,244**
Cda2-P245L	GVTPQCWRPPYGDVDNRVRMIAEGLNLTTI	103	81
Cda2-P270L	NLTTIIWSDDTDDWAAGTNGVTEQDVTNNY	13	27
**Cda2-P295L**	VTNNYQSVIDKAGNGTYTTHGPVVLNHELT	8	**1,059**
**Cda2-P320L**	NHELTNYTMSVFMTMFPKIKSAFNYIVPIC	10	**341**
**Cda2-P345L**	IVPICTAYNITQPYAESNITCPNFETYISG	10	**861**

Table S1. **Identification of DRB1*0401 and IAb epitopes of Cda2.** Screening for DRB1*0401 and IAb epitopes of Cda2 with >30 aa peptides and 30 aa overlapping peptides as stimulants of splenocytes from DR4 and C57BL/6 mice vaccinated with Cda2 recombinant protein. Stimulatory peptides are highlighted in red. IFNγ values are presented as the average of 3–4 mice per experiment.

Table S2

30 aa Peptide	Sequence	DR4 IFNγ (pg/mL)	C57BL/6 IFNγ (pg/mL)
Cda1-P20L	QTTGTSVDSSILTKTADSTGPSGFSIPALS	25	5
Cda1-P45L	IPALSELTSGAPTDSTVALYSTFAAGATPT	20	5
Cda1-P70L	GATPTVSGAPVLPTSALTIADYPALDVTPP	14	11
Cda1-P95L	DVTPPTNSSLVKDWMAKIDLSKVPSYNVTT	8	5
**Cda1-P120L**	YNVTTGDCSTDAAAISDGRCWWTCGGCTRE	8	**5,374**
Cda1-P145L	GCTRETDIVECPDKNVWGLSYDDGPSPFTP	9	5
Cda1-P170L	SPFTPLLIDYLQEKNIKTTFFVVGSRVLSR	10	5
Cda1-P195L	RVLSRPEMLQTEYMSGHQISIHTWSHPALT	6	5
Cda1-P220L	HPALTTLTNEEIVAELGWTMKVIKDTLGVT	7	5
Cda1-P245L	TLGVTPNTFRPPYGDIDDRVRAIAAQMGLT	8	9
**Cda1-P270L**	QMGLTPVIWTSYTDGSTTVNFDTNDWHISG	5	**1,466**
Cda2-P295L	WHISGGTATGASSYETFEKILTEYAPKLDT	132	5
**Cda1-P320L**	PKLDTGFITLEHDIYQQSVDLAVGYILPQV	**11,138**	5
**Cda1-P345L**	ILPQVLANGTYQLKSIINCLGKDTSEAYIE	**13,664**	5

Table S2. **Identification of DRB1*0401 and IAb epitopes of Cda1.** Screening for DRB1*0401 and IAb epitopes of Cda1 with 30 aa overlapping peptides as stimulants of splenocytes from DR4 and C57BL/6 mice vaccinated with Cda1 recombinant protein. Stimulatory peptides are highlighted in red. IFNγ values are presented as the average of 3–4 mice per experiment.

Table S3A

Cda2 DRB1*0401 epitopes					
Cda2 P36 epitope		Cda2 P52 epitope		Cda2 P74 epitope	
Unstim vs.	P value	Unstim vs.	P value	Unstim vs.	P value
P31	>0.9999	P47	>0.9999	P68	>0.9999
P32	0.9996	P48	>0.9999	P69	>0.9999
P33	0.9752	P49	0.8071	P70	0.2007
P34	0.1058	P50	0.7319	P71	0.0020
P35	0.0101	P51	0.0322	P72	<0.0001
P36	0.0024	P52	0.0140	P73	<0.0001
P37	0.0143	P53	0.0183	P74	<0.0001
P38	0.0795	P54	0.0930	P75	<0.0001
P39	0.0184	P55	0.7622	P76	<0.0001
P40	0.9999	P56	>0.9999	P77	0.9186
P41	>0.9999			P78	>0.9999
				P79	>0.9999

Table S3B

Cda2 IAb epitopes					
Cda2 P36 epitope		Cda2 P60 epitope		Cda2 P69 epitope	
Unstim vs.	P value	Unstim vs.	P value	Unstim vs.	P value
P31	>0.9999	P54	0.9999	P64	>0.9999
P32	>0.9999	P55	0.9999	P65	>0.9999
P33	0.2293	P56	0.1264	P66	0.5068
P34	<0.0001	P57	0.0004	P67	0.0549
P35	<0.0001	P58	<0.0001	P68	0.0548
P36	<0.0001	P59	<0.0001	P69	0.0809
P37	<0.0001	P60	0.0128	P70	0.1862
P38	0.9997	P61	0.9930	P71	0.9997
P39	0.9997	P62	>0.9999	P72	>0.9999
		P63	0.9999		

Table S3B (continued)

Cda2 IAb epitopes					
Cda2 P217 epitope		Cda2 P231 epitope		Cda2 P308 epitope	
Unstim vs.	P value	Unstim vs.	P value	Unstim vs.	P value
P211	>0.9999	P227	>0.9999	P305	>0.9999
P212	>0.9999	P228	0.9996	P306	0.0786
P213	0.9994	P229	0.7637	P307	0.5520
P214	0.0243	P230	0.7580	P308	0.5269
P215	<0.0001	P231	0.0009	P309	0.4402
P216	<0.0001	P232	0.4199	P310	0.6011
P217	<0.0001	P233	0.9968	P311	0.4961
P218	<0.0001	P234	0.9999	P312	0.7265
P219	<0.0001			P313	0.9994
P220	0.2551			P314	0.9997
P221	0.9811				
P222	0.9844				
P223	>0.9999				
P224	>0.9999				

Table S3. Statistical analysis of Cda2 epitope mapping data shown in Figures 2 and 3. Table S3A shows the analysis for the DRB1*0401 epitopes (DR4 mice) whereas **Table S3B** shows the analysis for IAb epitopes (C57BL/6 mice). P values were calculated using ordinary one-way ANOVAs and adjusted for multiple pair-wise comparisons using the Dunnett's multiple comparison test. Comparisons are of IFNγ concentrations in the supernatants of splenocytes from immunized mice that were either left unstimulated (Unstim) or stimulated with the indicated 15 aa peptides. Results are from two independent experiments, each with 3-4 mice per group (n=7-8/peptide).

Table S4A

Cda1 DRB1*0401 epitopes			
**Cda1 P324 epitope**		**Cda1 P354 epitope**	
Unstim vs.	P Value	Unstim vs.	P Value
P319	>0.9999	P349	>0.9999
P320	>0.9999	P350	>0.9999
P321	0.0077	P351	0.7971
P322	<0.0001	P352	<0.0001
P323	<0.0001	P353	<0.0001
P324	<0.0001	P354	<0.0001
P325	<0.0001	P355	<0.0001
P326	0.0299	P356	<0.0001
P327	0.7309	P357	0.0313
P328	>0.9999	P358	0.9999
P329	>0.9999	P359	>0.9999

Table S4B

Cda1 IAb epitopes			
**Cda1 P138 epitope**		**Cda1 P269 epitope**	
Unstim vs.	P Value	Unstim vs.	P Value
P133	>0.9999	P266	>0.9999
P134	0.9996	P267	>0.9999
P135	<0.0001	P268	0.6684
P136	<0.0001	P269	0.1168
P137	<0.0001	P270	0.0136
P138	<0.0001	P271	0.1367
P139	<0.0001	P272	0.3531
P140	0.9639	P273	0.4636
P141	>0.9999	P274	0.9991
P142	>0.9999	P275	0.9992

Table S4. Statistical analysis of Cda1 epitope mapping data shown in Figures 4 and 5. Table S4A shows the analysis for the DRB1*0401 epitopes (DR4 mice) whereas **Table S4B** shows the analysis for IAb epitopes (C57BL/6 mice). P values were calculated using ordinary one-way ANOVAs and adjusted for multiple pair-wise comparisons using the Dunnett's multiple comparison test. Comparisons are of IFNγ concentrations in the supernatants of splenocytes from immunized mice that were either left unstimulated (Unstim) or stimulated with the indicated 15 aa peptides. Results are from two independent experiments, each with 3-4 mice per group (n=7-8/peptide).

Table S5

	$CD3^+$ (%)		$CD3^+CD8^+$ (%)		$CD3^+CD4^+$ (%)	
**CDA1**	**Mock-depleted**	**CD4-depleted**	**Mock-depleted**	**CD4-depleted**	**Mock-depleted**	**CD4-depleted**
**BL6-M1**	33.80	22.35	40.45	84.40	50.50	0.20
**BL6-M2**	54.60	35.25	39.95	87.45	53.25	0.17
**BL6-M3**	44.20	21.20	33.20	88.65	61.30	0.28
**BL6-M4**	56.10	22.30	40.05	88.40	53.85	0.32
**BL6-M5**	22.70	17.35	41.55	89.85	51.20	0.14
**BL6-M6**	33.50	24.95	38.35	90.90	56.30	0.15
**BL6-M7**	28.60	18.70	38.15	90.25	56.00	0.20
**BL6-M8**	27.65	21.65	41.05	89.80	52.65	0.26
**MEAN ± SD**	**38 ± 12.6**	**23 ± 5.5**	**39 ± 2.7**	**89 ± 2.1**	**54 ± 3.5**	**0.2 ± 0.07**
**DR4-M1**	61.75	16.00	9.49	72.30	85.30	0.21
**DR4-M2**	45.00	8.88	10.70	68.90	83.10	0.38
**DR4-M3**	47.50	10.30	10.35	72.75	84.30	0.36
**DR4-M4**	51.80	13.10	10.40	71.70	84.10	0.82
**DR4-M5**	36.65	9.21	10.80	72.50	82.60	0.39
**DR4-M6**	39.85	8.95	10.80	71.90	83.30	0.40
**DR4-M7**	40.40	10.65	10.40	72.15	83.45	0.42
**DR4-M8**	41.20	10.15	11.40	74.10	82.10	1.22
**MEAN ± SD**	**46 ± 8.1**	**10.9 ± 2.5**	**10.5 ± 0.5**	**72 ± 1.5**	**84 ± 1.0**	**0.5 ± 0.33**

	$CD3^+$ (%)		$CD3^+CD8^+$ (%)		$CD3^+CD4^+$ (%)	
**CDA2**	**Mock-depleted**	**CD4-depleted**	**Mock-depleted**	**CD4-depleted**	**Mock-depleted**	**CD4-depleted**
**BL6-M1**	43.45	28.85	40.05	86.70	53.20	0.41
**BL6-M2**	48.35	32.55	37.15	86.60	55.80	0.35
**BL6-M3**	50.70	32.95	39.50	87.60	53.85	0.55
**BL6-M4**	39.05	21.60	42.20	83.65	48.65	0.89
**BL6-M5**	29.00	14.35	37.90	84.45	54.25	0.32
**BL6-M6**	27.75	13.30	39.20	85.40	52.75	0.30
**BL6-M7**	22.55	12.25	39.25	85.35	52.60	0.42
**BL6-M8**	32.15	15.80	38.10	85.85	53.80	0.53
**MEAN ± SD**	**37 ± 10.3**	**21 ± 8.8**	**39 ± 1.6**	**86 ± 1.3**	**53 ± 2.1**	**0.5 ± 0.19**
**DR4-M1**	43.50	9.25	10.75	74.15	84.20	0.77
**DR4-M2**	40.55	9.12	9.67	73.80	85.55	0.33
**DR4-M3**	43.80	9.65	9.65	71.30	85.05	0.27
**DR4-M4**	42.05	8.67	10.35	73.95	84.85	1.12
**DR4-M5**	44.10	14.60	13.10	66.90	79.00	0.97
**DR4-M6**	55.75	18.55	12.20	68.60	80.95	0.36
**DR4-M7**	50.40	18.45	12.95	69.00	80.25	2.50
**DR4-M8**	44.25	14.25	11.50	66.10	81.10	1.93
**MEAN ± SD**	**46 ± 5.0**	**13 ± 4.2**	**11 ± 1.4**	**70 ± 3.3**	**83 ± 2.6**	**1.0 ± 0.81**

Table S5. Frequencies of $CD3^+$, $CD3^+CD8^+$, and $CD3^+CD4^+$ populations in splenocytes with and without $CD4^+$ T cell depletion. C57BL/6 (BL6) and DR4 mice were immunized with either

Cda1 or Cda2 protein. Splenocyte preparations were made and either mock-depleted or depleted of $CD4^+$ T cells (CD4-depleted) by passage over a magnetic bead column. Samples were analyzed by flow cytometry to assess T cell populations in the mock-depleted and CD4-depleted groups. Percentages are presented as the average of duplicate measurements for each mouse splenocyte preparation. The percentage of $CD3^+$ T cells was determined based on the gate for the $CD45^+$ population. The percentages of $CD4^+$ and $CD8^+$ T cells were determined based on the gate for the $CD3^+$ T cell population. The gating strategy is shown in Figure S2. Two independent experiments were performed, each including four mice, totaling eight mice per group. Individual mice are labeled M1–M8.

Table S6

Cda2-P36 epitope (DRB1*0401 and IAb)

P#	DRB1* 01:01	DRB1* 03:01	DRB1* 04:01	DRB1* 04:05	DRB1* 07:01	DRB1* 08:02	DRB1* 09:01	DRB1* 11:01	DRB1* 12:01	DRB1* 13:02	DRB1* 15:01
P32	1071	7648	4619	2820	2897	1454	2501	1178	4381	3663	1239
P33	333	7047	138	262	1194	816	422	804	4329	2431	1065
P34	136	6741	62	185	1001	546	305	709	4895	2284	1248
P35	75	6223	45	147	845	382	236	572	5339	2069	1265
**P36**	**52**	**3695**	**38**	**131**	**757**	**301**	**201**	**467**	**5492**	**1577**	**1489**
P37	47	2114	31	114	707	312	178	516	5563	1086	2193
P38	102	1794	58	231	1240	672	351	1154	7407	1302	3374
P39	396	1676	374	1200	2262	2501	1211	4987	8146	1440	4599

Cda2-P52 epitope (DRB1*0401)

P#	DRB1* 01:01	DRB1* 03:01	DRB1* 04:01	DRB1* 04:05	DRB1* 07:01	DRB1* 08:02	DRB1* 09:01	DRB1* 11:01	DRB1* 12:01	DRB1* 13:02	DRB1* 15:01
P49	3017	2640	1153	2757	6669	7227	6375	11644	11515	4840	9191
P50	1238	3002	409	1415	4279	3812	3870	7377	6393	3753	5998
P51	670	3687	239	1056	3575	2466	3200	5286	5151	3058	4333
**P52**	**789**	**4284**	**284**	**1254**	**4531**	**2692**	**3753**	**5801**	**5870**	**3879**	**4977**
P53	789	4949	380	1374	2157	2980	1867	5914	4137	3698	1893
P54	911	6744	796	2024	2017	3938	1399	6899	4338	4443	1513
P55	1266	10719	2870	2989	2255	5318	1448	7554	4733	5358	1506

Cda2-P59 epitope (IAb)

P#	DRB1* 01:01	DRB1* 03:01	DRB1* 04:01	DRB1* 04:05	DRB1* 07:01	DRB1* 08:02	DRB1* 09:01	DRB1* 11:01	DRB1* 12:01	DRB1* 13:02	DRB1* 15:01
P56	334	12452	1327	700	206	4406	128	6157	5421	3828	1105
P57	96	9793	543	388	106	2858	67	4185	5202	2634	1013
P58	58	8904	406	281	96	1566	59	1959	4644	2567	1134
**P59**	**47**	**8852**	**331**	**255**	**104**	**932**	**61**	**885**	**5218**	**2595**	**1517**
P60	45	7049	278	223	107	628	63	498	4021	2398	1672
P61	80	7789	369	296	187	697	97	572	3470	2931	2368

Cda2-P68 epitope (IAb)

P#	DRB1* 01:01	DRB1* 03:01	DRB1* 04:01	DRB1* 04:05	DRB1* 07:01	DRB1* 08:02	DRB1* 09:01	DRB1* 11:01	DRB1* 12:01	DRB1* 13:02	DRB1* 15:01
P66	101	1803	258	130	150	543	168	1195	631	252	73
P67	93	1470	162	105	136	358	144	800	547	199	50
**P68**	**121**	**1895**	**153**	**107**	**181**	**331**	**168**	**714**	**611**	**275**	**49**
P69	167	2924	237	144	271	439	255	894	842	492	63
P70	262	5622	560	299	676	784	565	1409	1400	1398	125

Cda2-P74 epitope (DRB1*0401)

P#	DRB1* 01:01	DRB1* 03:01	DRB1* 04:01	DRB1* 04:05	DRB1* 07:01	DRB1* 08:02	DRB1* 09:01	DRB1* 11:01	DRB1* 12:01	DRB1* 13:02	DRB1* 15:01
P70	262	5622	560	299	676	784	565	1409	1400	1398	125
P71	192	6711	515	425	57	801	148	1715	2118	461	463
P72	115	5172	238	321	56	515	124	1177	2562	373	427
P73	122	4891	194	320	74	478	133	1130	2801	474	465
**P74**	**109**	**4664**	**166**	**309**	**81**	**413**	**138**	**1010**	**2750**	**468**	**440**
P75	211	6768	249	476	174	686	242	1942	4867	930	1053
P76	265	8799	392	621	229	1027	347	2947	6120	1200	1630
P77	628	12781	1162	1419	1082	2578	990	6190	9792	3248	4990

Cda1-P324 epitope (DRB1*0401)

P#	DRB1* 01:01	DRB1* 03:01	DRB1* 04:01	DRB1* 04:05	DRB1* 07:01	DRB1* 08:02	DRB1* 09:01	DRB1* 11:01	DRB1* 12:01	DRB1* 13:02	DRB1* 15:01
P321	705	1736	782	497	1823	3074	1456	2059	1082	2694	1669
P322	635	1271	257	403	2059	2333	1705	1626	984	2018	1298
P323	544	740	176	385	2450	1903	1885	1386	947	1248	1104
**P324**	**356**	**415**	**122**	**293**	**2126**	**1412**	**1424**	**1047**	**759**	**815**	**712**
P325	501	445	175	359	3095	1905	1844	1495	970	1134	968
P326	492	451	438	595	2014	3001	2013	2887	1498	992	1300
P327	304	846	1136	1230	1974	4283	2207	6036	2957	1467	1873

Cda1-P354 epitope (DRB1*0401)

P#	DRB1* 01:01	DRB1* 03:01	DRB1* 04:01	DRB1* 04:05	DRB1* 07:01	DRB1* 08:02	DRB1* 09:01	DRB1* 11:01	DRB1* 12:01	DRB1* 13:02	DRB1* 15:01
P351	103	5106	324	264	365	529	643	602	1519	644	557
P352	44	3767	91	123	272	195	453	186	959	434	169
P353	36	3592	67	105	320	161	459	147	924	434	140
**P354**	**33**	**3364**	**62**	**105**	**368**	**150**	**479**	**131**	**897**	**409**	**124**
P355	44	3564	92	152	529	227	664	196	1121	594	183
P356	74	4318	211	334	994	453	1143	381	1857	1115	405
P357	152	5133	1502	1558	2191	1160	1971	666	2867	1889	1081

Table S6. In silico predictions of binding affinities to 11 most common human HLA-DRB1* alleles for the seven epitopes found in the Cda2/1 hybrid protein. The predicted IC50 values (nM) of each 15 aa peptide comprising the epitope are shown for the 11 most common human HLA-DRB1* alleles. Each 15 aa peptide is identified by its P# listed in the first column. Below each MHC-II allele are the predicted IC50 values of each peptide comprising the epitope. Predictions were made using the IEDB tool, NetMHCIIpan 4.1 BA. Highlighted in gray are the IC50 values that are <1,000 nM. See Figure 7A-D for details regarding the seven epitopes.
